# Supplementary material for: Analysis of early intermediate states of the nitrogenase reaction by regularization of EPR spectra
Source: Nat Commun. 2024 May 13;15:4041. doi: 10.1038/s41467-024-48271-8 (PMC11091149; doi:10.1038/s41467-024-48271-8)
Supplement: Supplementary file 1 — Supplementary Information [file 41467_2024_48271_MOESM1_ESM.pdf]

Analysis of early intermediate states of the nitrogenase reaction by regularization of EPR spectra

Lorenz Heidinger[1,3], Kathryn Perez[2], Thomas Spatzal[2], Oliver Einsle[3], Stefan Weber[1], Douglas Rees[2]*, Erik Schleicher[1]*

[1]Institut für Physikalische Chemie, Albert-Ludwigs-Universität Freiburg, 79104 Freiburg, Germany.
[2]Howard Hughes Medical Institute (HHMI), California Institute of Technology, Division of Chemistry and Chemical Engineering, Pasadena, CA 91125, USA.
[3]Institut für Biochemie, Albert-Ludwigs-Universität Freiburg, 79104 Freiburg, Germany.

- Supplementary Information -

Content

Supplementary Figures

Supplementary Tables

Supplementary Notes

Supplementary References

Supplementary Figures:

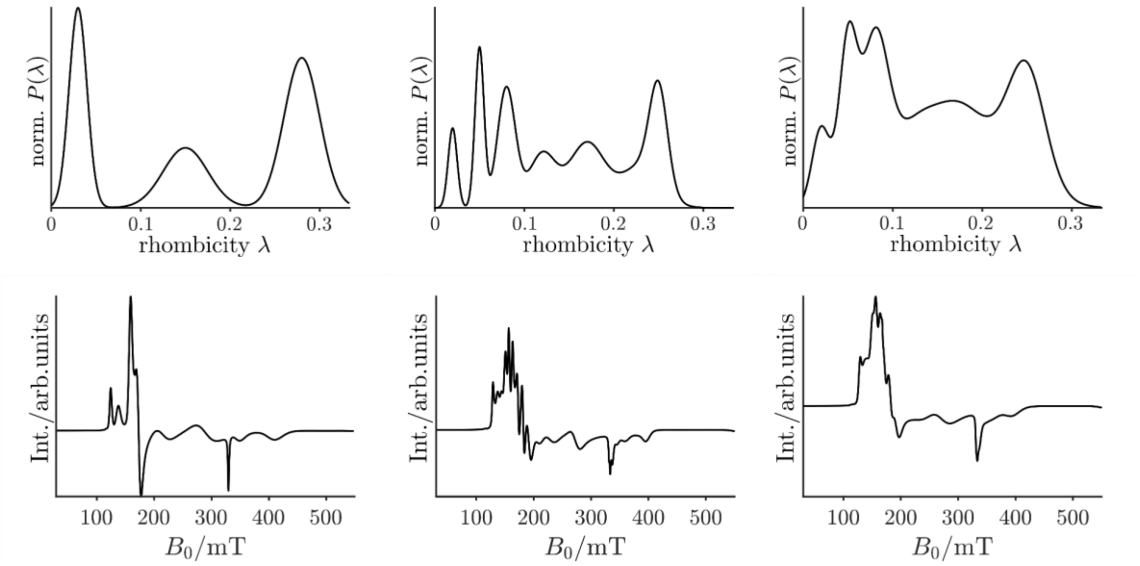

Supplementary Figure 1. Probability distributions (top) and corresponding cw-EPR spectra (bottom) of the three calculated model datasets model 1 (left), model 2 (center) and model 3 (right).

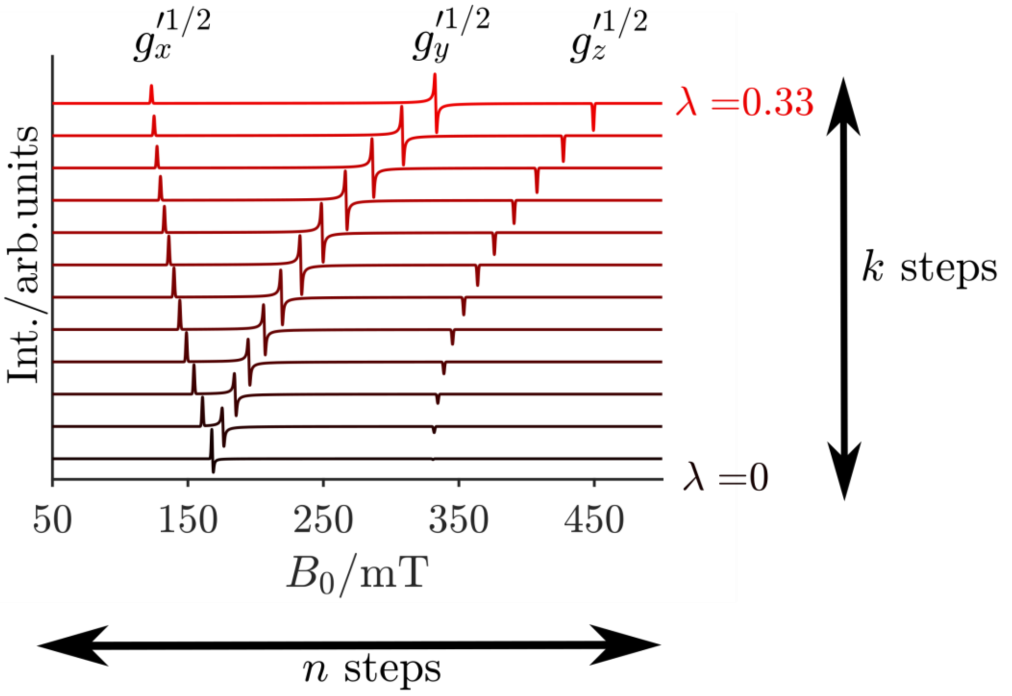

Supplementary Figure 2. Exemplary representation of normalized EPR spectra of kernel A. To generate the kernel, the magnetic field axis is discretized into n steps and the parameter axis is discretized into k steps. The color gradient shows exemplary cw-EPR spectra from $\lambda = 0$ (black) to $\lambda = 0.33$ (red) in steps of 0.03. The following parameters were used: $S' = 1/2$, $g_x = g_y = 2.00$, $g_z = 2.03$, and an intrinsic linewidth of 1 mT. All simulations were performed using the EasySpin algorithm pepper[1].

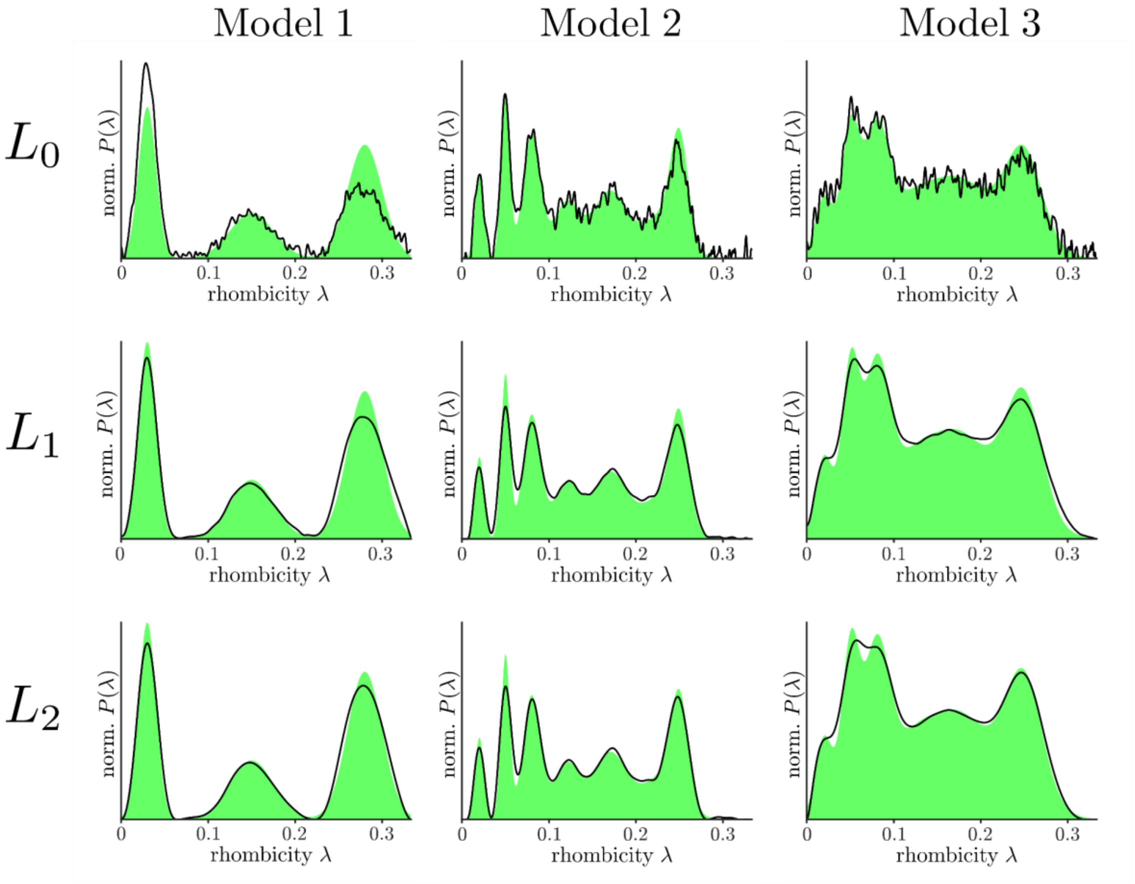

Supplementary Figure 3. Results of Tikhonov regularization of model 1, 2 and 3 using the different regularization operators L_0, L_1 and L_2. The theoretical distribution functions $P_{\mathrm{Theo}}(\lambda)$ are shown as green areas. The optimal results of the regularization $P(\lambda)$, using a Gaussian noise model with a S/N of 20, are depicted as black curves. α_{Opt} was determined using the GCV method.

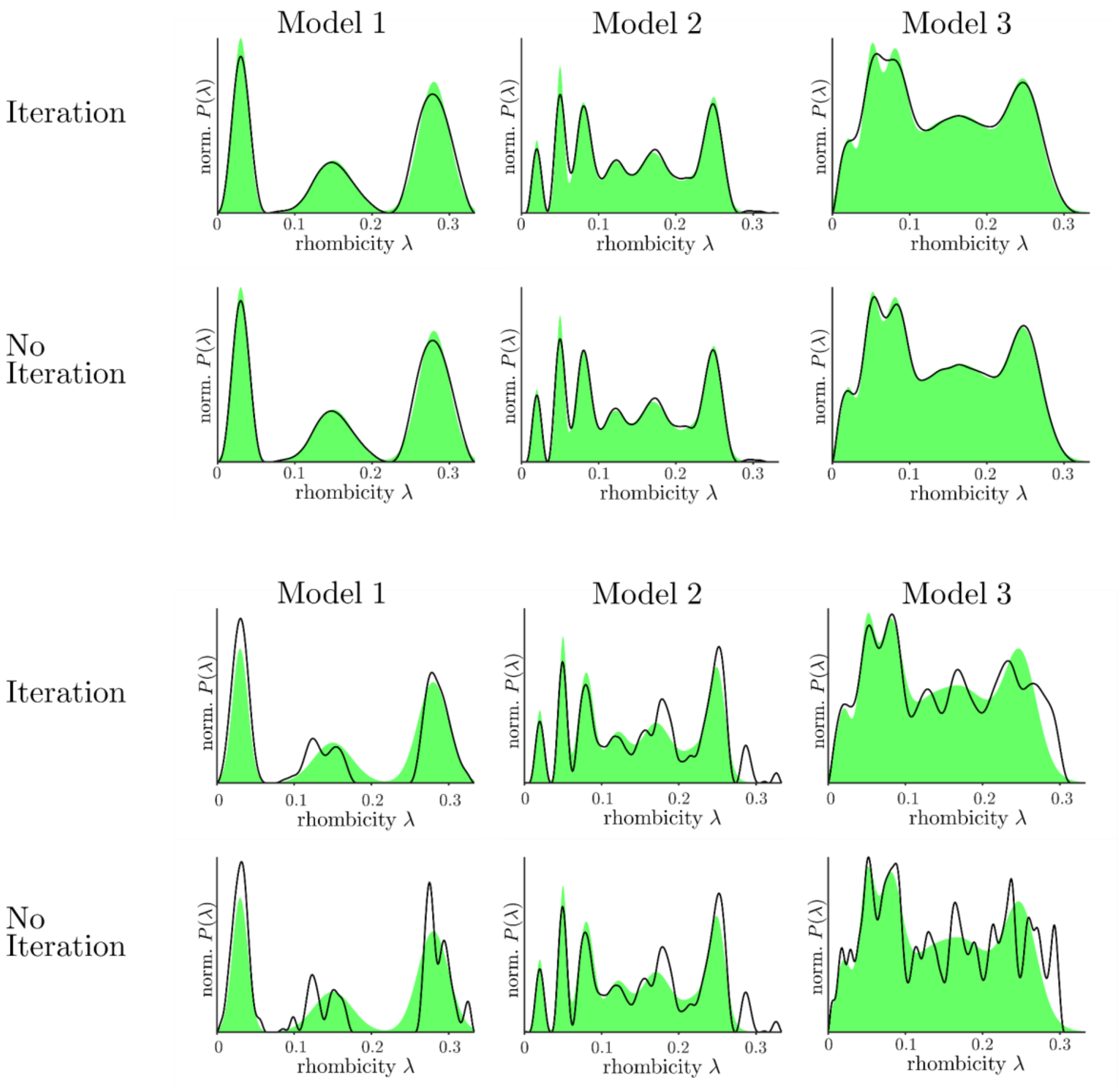

Supplementary Figure 4. Comparison of regularization results of models 1, 2 and 3 with and without Osher-Bregman iteration. Either a Gaussian noise model with a S/N of 20 (upper panels), or a $1/f$ noise model with a S/N of 20 (lower panels) were applied. L_2 was used as regularization operator, α_{Opt} was determined using the GCV method. $P_{\mathrm{Theo}}(\lambda)$ is shown as green areas, and $P(\lambda)$ as black curves.

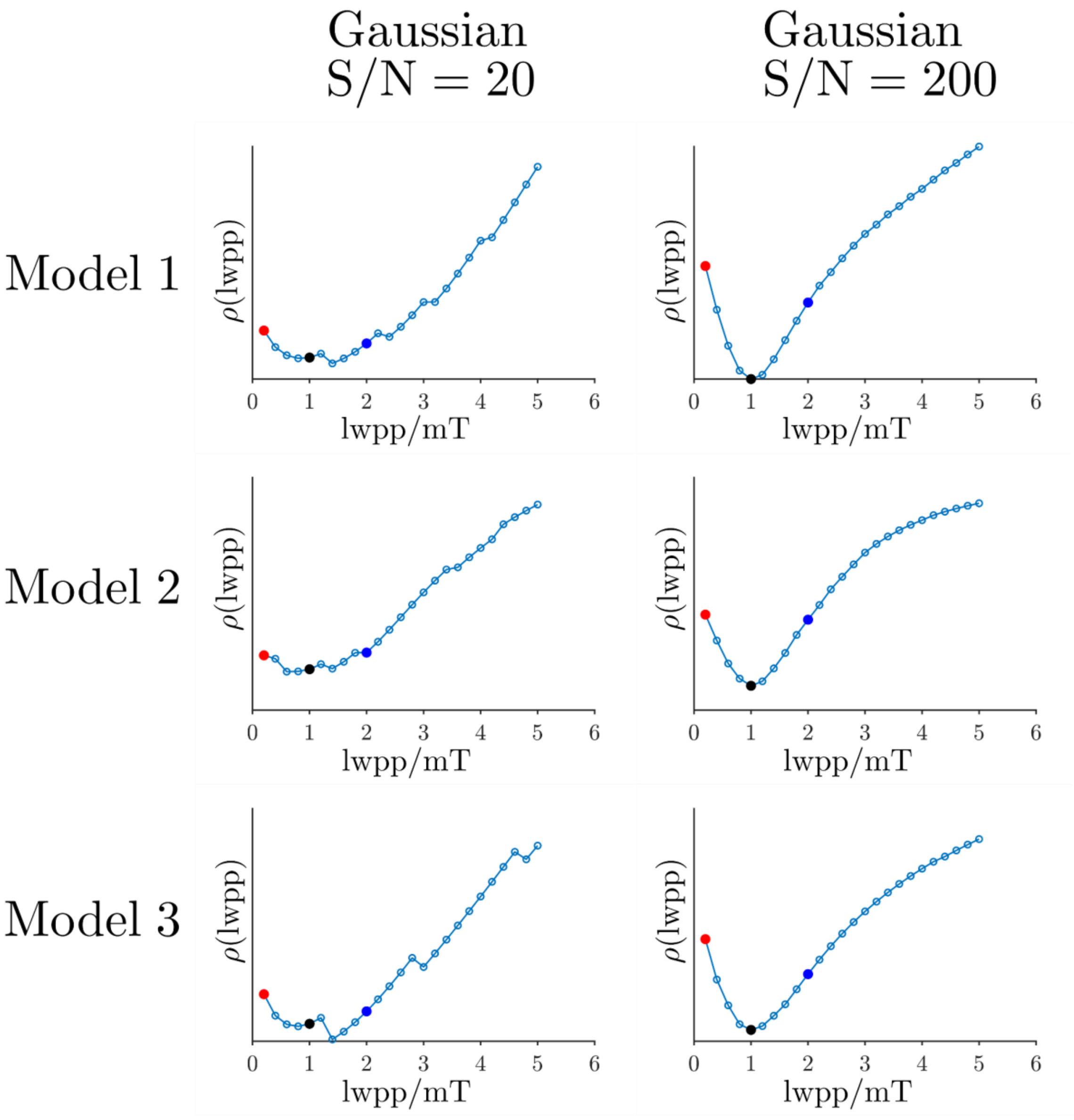

Supplementary Figure 5. ρ(lwpp) of models 1, 2 and 3 is plotted against lwpp. Each model was analyzed with a Gaussian noise model and a S/N of 20 and 200, respectively. The true intrinsic linewidth is lwpp $=$ 1 mT (black dot). Red and blue dots correspond to a linewidth of 0.2 mT and 2.0 mT. The corresponding probability distributions and spectra are plotted in Supplementary Figures 7, 9 and 10, and are compared with $P_{\mathrm{Theo}}(\lambda)$. Even a low S/N of 20 yields to a very good agreement between the minimum of ρ(lwpp) and the true linewidth.

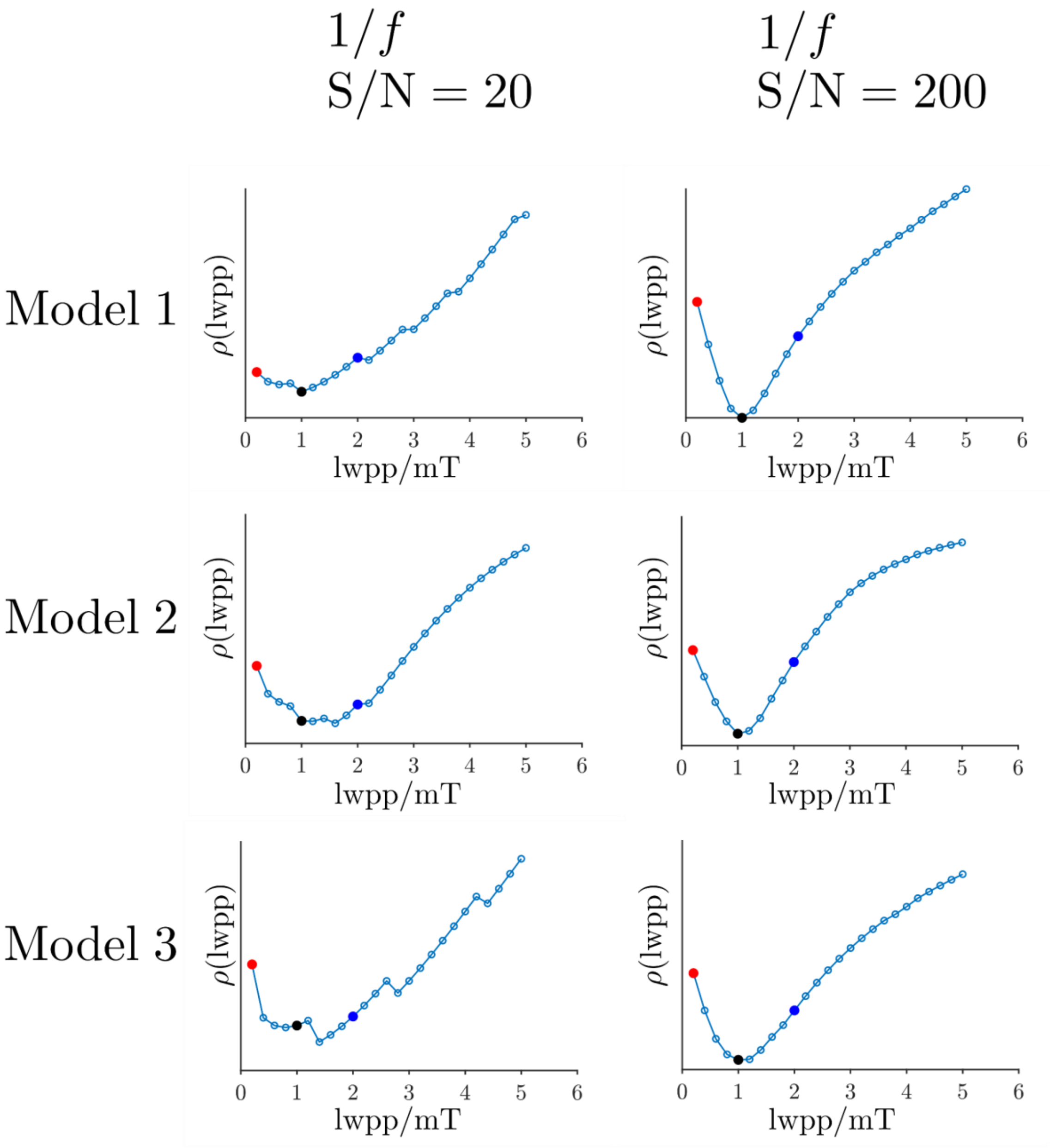

Supplementary Figure 6. $\rho(\text{lwpp})$ of models 1, 2 and 3 is plotted against lwpp. Each model was analyzed with a 1/f noise model and a S/N of 20 and 200, respectively. The true intrinsic linewidth is lwpp $= 1\,\text{mT}$ (black dot). Red and blue dots correspond to a linewidth of $0.2\,\text{mT}$ and $2.0\,\text{mT}$, respectively. The corresponding probability distributions and spectra are plotted in Supplementary Figures 7, 11 and 12, and are compared with $P_{\text{Theo}}(\lambda)$. Even a low S/N of 20 yields to a very good agreement between the minimum of $\rho(\text{lwpp})$ and the true linewidth.

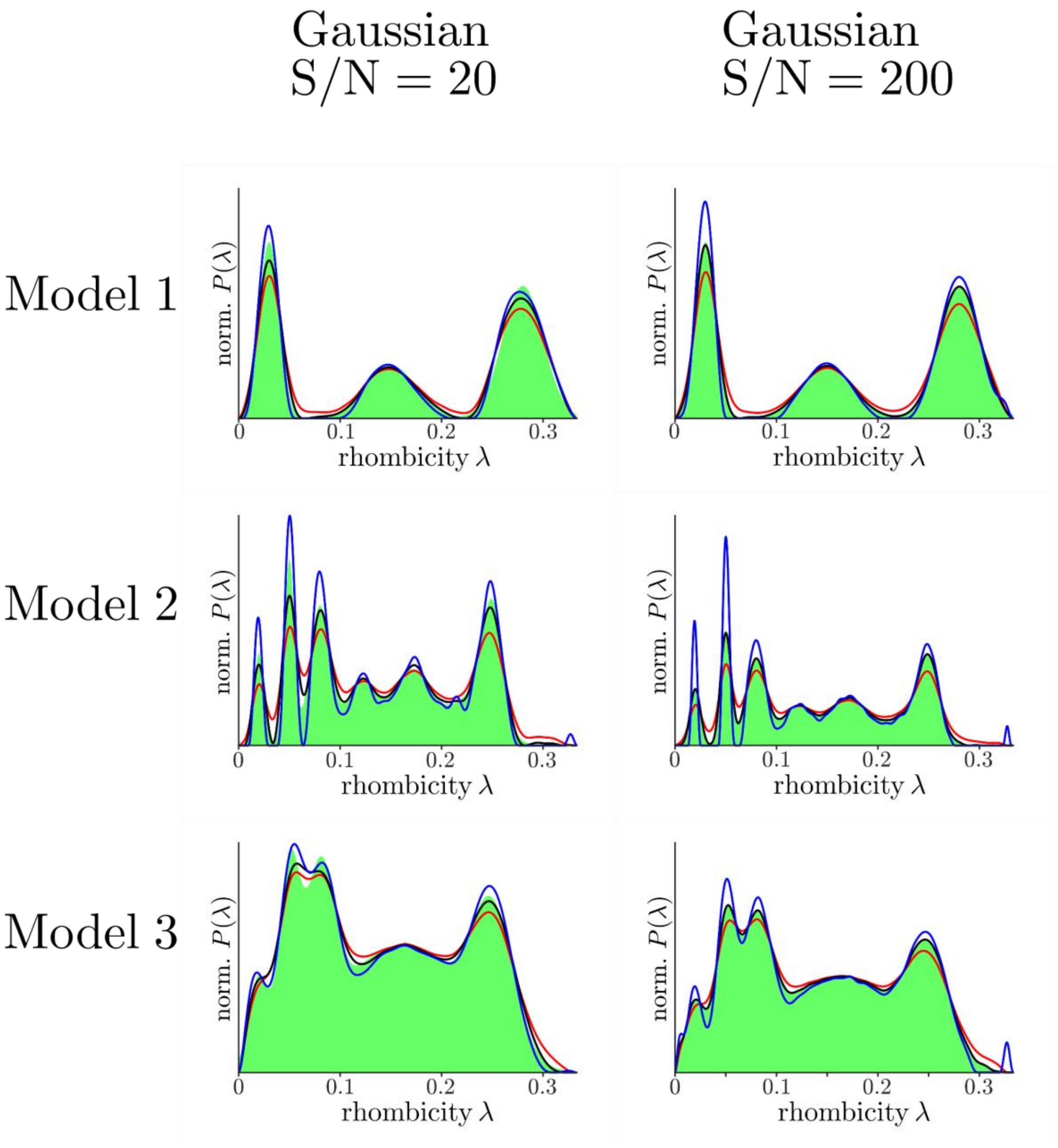

Supplementary Figure 7. Normalized probability distributions of models 1, 2, and 3. Each model was analyzed with a Gaussian noise and a S/N of 20 and 200, respectively. The black curve shows the result of the regularization for the true linewidth (1.0 mT), the red curve for a linewidth that is too small (0.2 mT) and the blue curve for a linewidth that is too large (2.0 mT). The theoretical distribution $P_{\mathrm{Theo}}(\lambda)$ is shown as green areas.

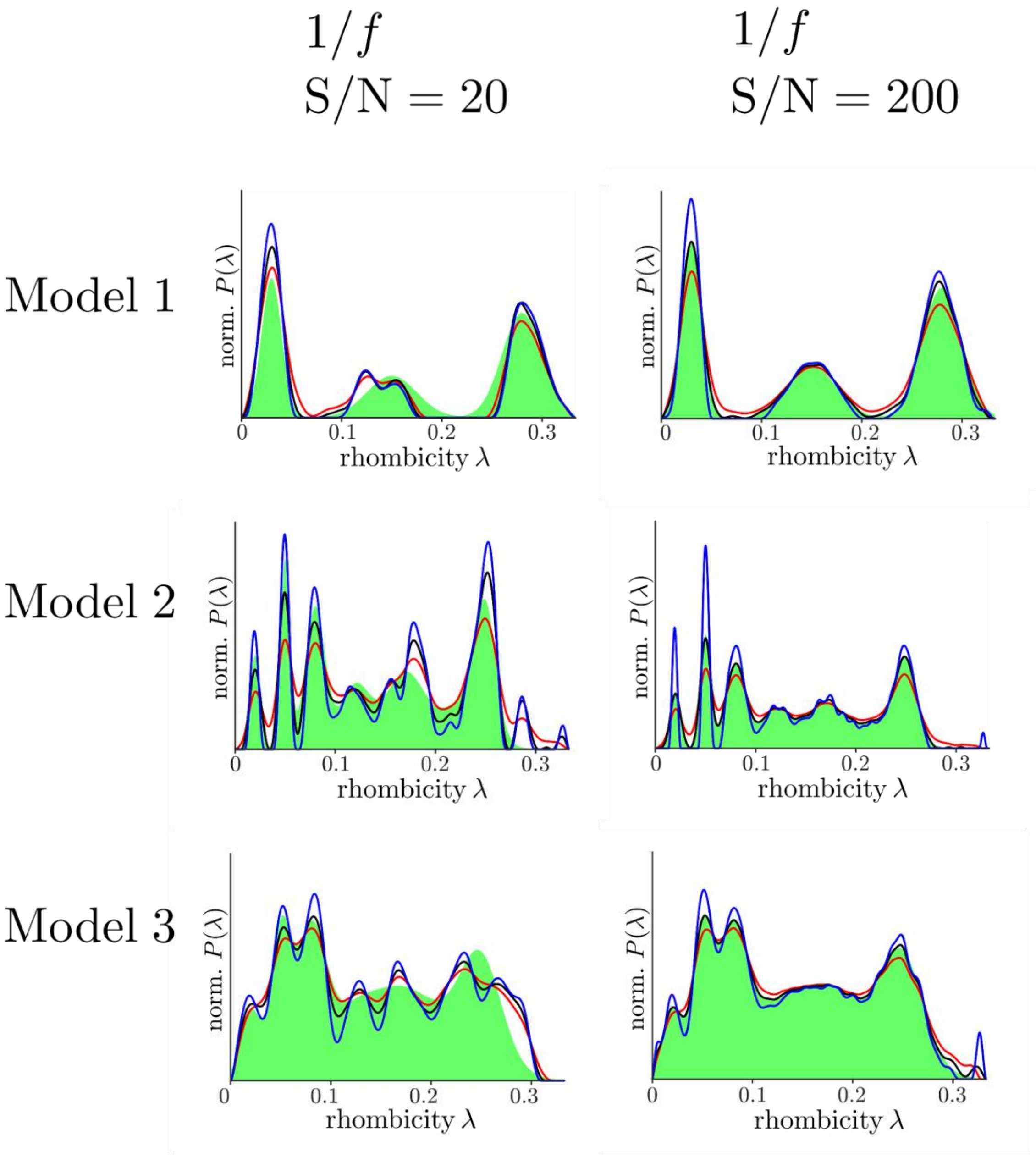

Supplementary Figure 8. Normalized probability distribution of models 1, 2, and 3. Each model was analyzed with a 1/f noise and a S/N of 20 and 200, respectively. The black curve shows the result of the regularization for the true linewidth (1.0 mT), the red curve for a linewidth that is too small (0.2 mT) and the blue curve for a linewidth that is too large (2.0 mT). The theoretical distribution $P_{\mathrm{Theo}}(\lambda)$ is shown as green areas.

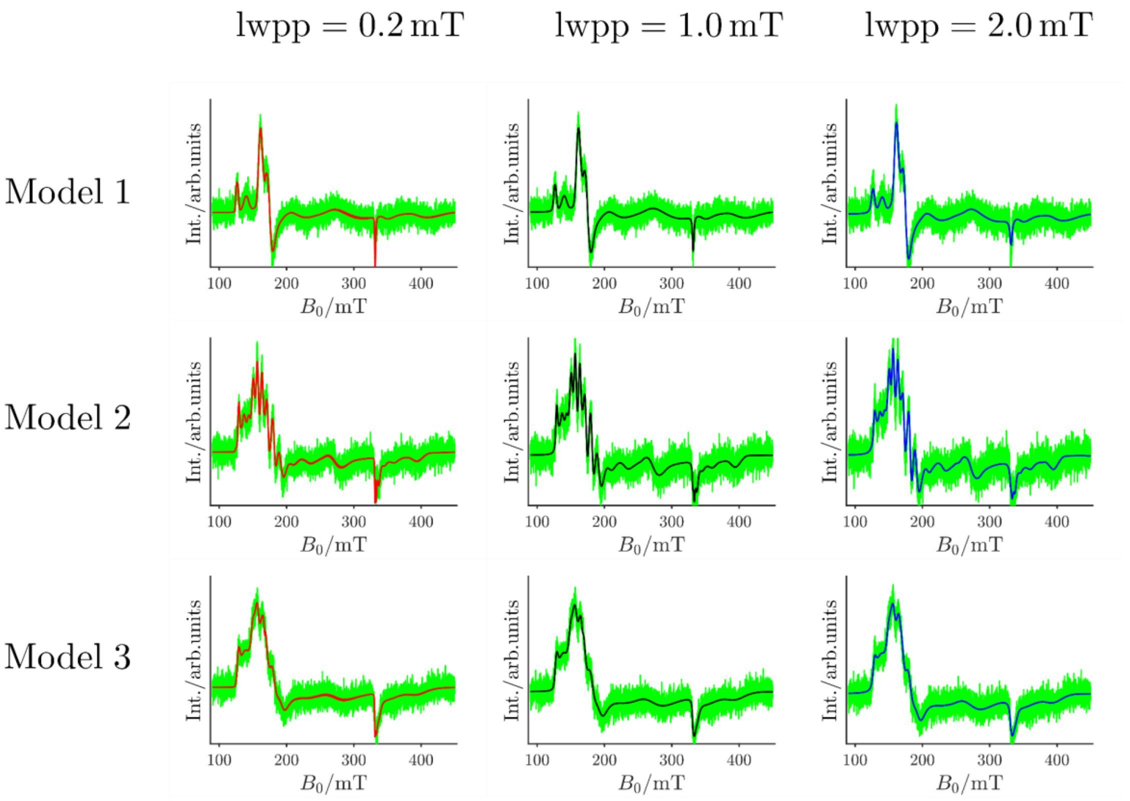

Supplementary Figure 9. Comparison of the calculated cw-EPR spectra of models 1, 2 and 3 with the results of the regularization. The correct spectra were calculated with a Gaussian noise ($S/N = 20$) and a linewidth of 1.0 mT (green curves). The black spectra display the regularization result for a correct linewidth of 1.0 mT, the red spectra for a too small linewidth of 0.2 mT and the blue spectra for a too large linewidth of 2.0 mT.

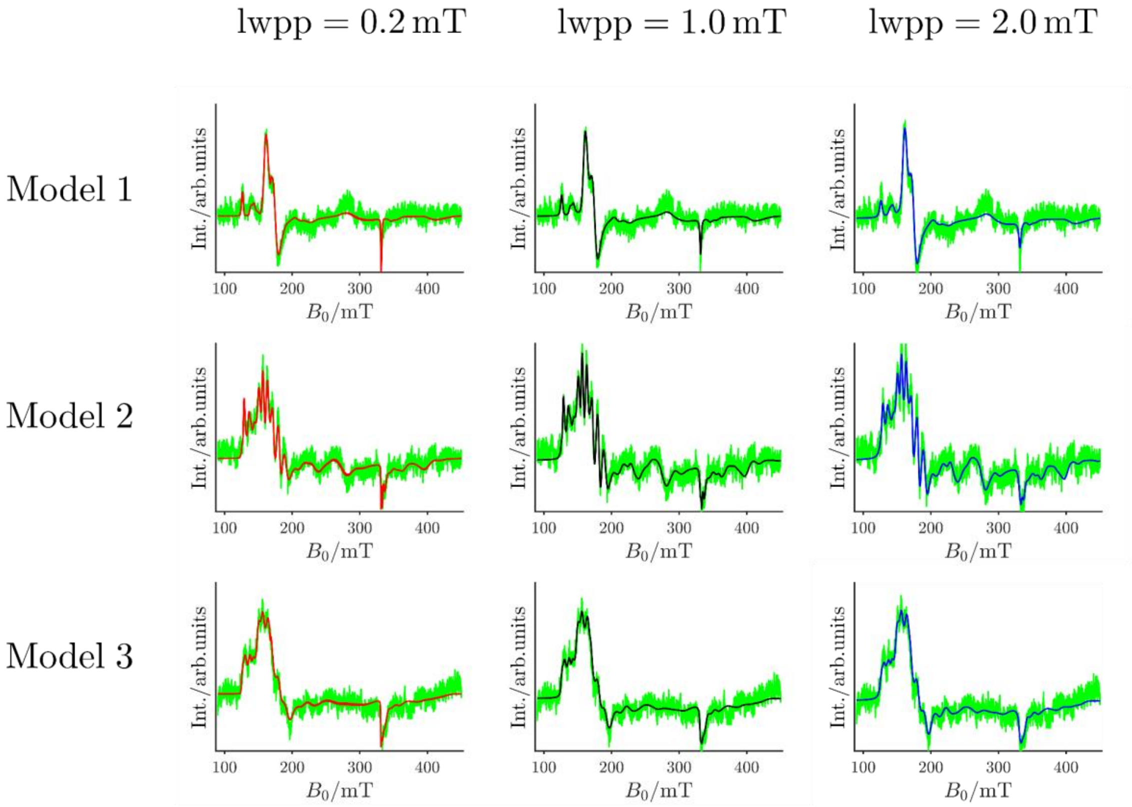

Supplementary Figure 10. Comparison of the calculated cw-EPR spectra of models 1, 2 and 3 with the results of the regularization. The correct spectra were calculated with a Gaussian noise ($S/N = 200$) and a linewidth of 1.0 mT (green curves). The black spectra display the regularization result for a correct linewidth of 1.0 mT, the red spectra for a too small linewidth of 0.2 mT and the blue spectra for a too large linewidth of 2.0 mT.

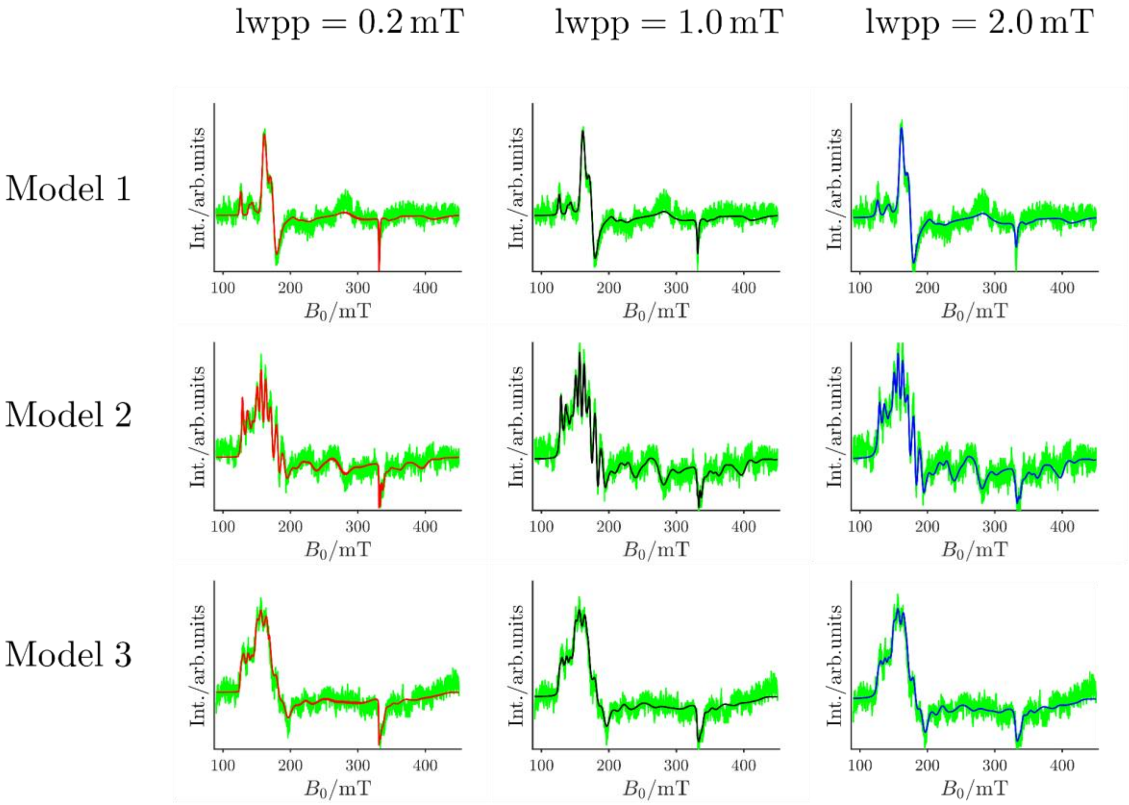

Supplementary Figure 11. Comparison of the calculated cw-EPR spectra of models 1, 2 and 3 with the results of the regularization. The correct spectra were calculated with a $1/f$ noise ($\mathrm{S/N} = 20$) and a linewidth of 1.0 mT (green curves). The black spectra display the regularization result for a correct linewidth of 1.0 mT, the red spectra for a too small linewidth of 0.2 mT and the blue spectra for a too large linewidth of 2.0 mT.

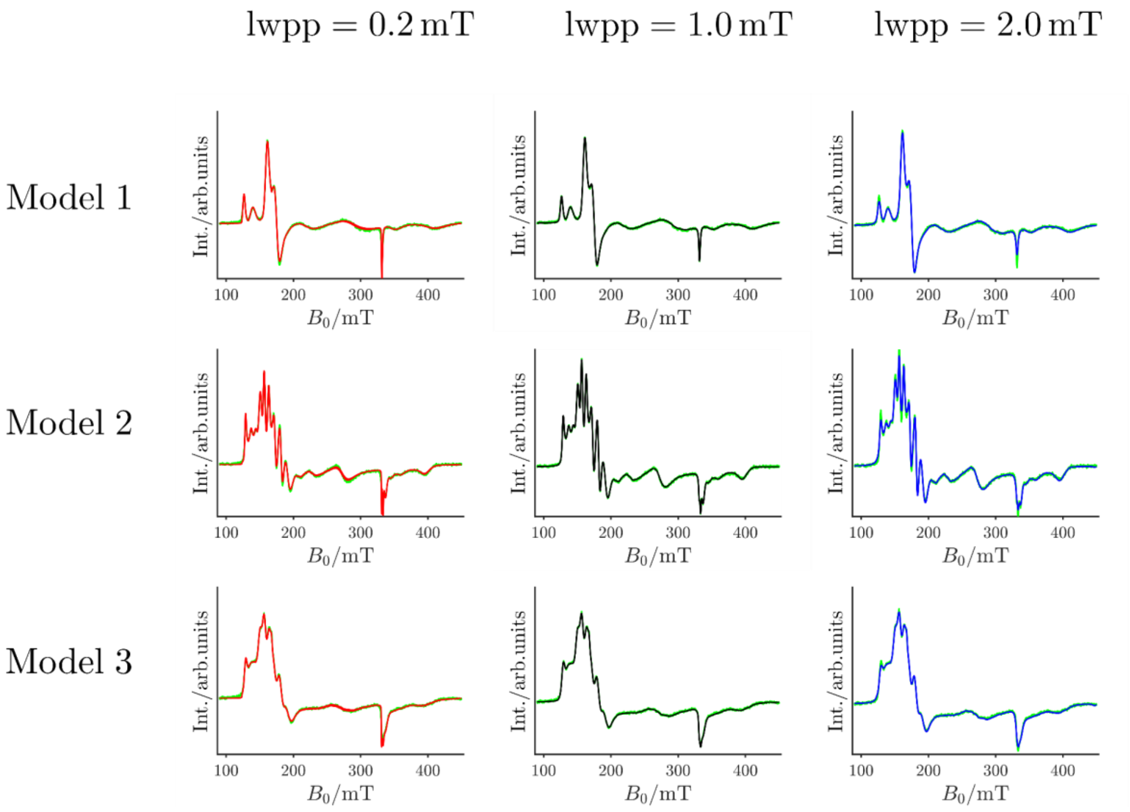

Supplementary Figure 12. Comparison of the calculated cw-EPR spectra of models 1, 2 and 3 with the results of the regularization. The correct spectra were calculated with a $1/f$ noise ($\mathrm{S/N} = 200$) and a linewidth of 1.0 mT (green curves). The black spectra display the regularization result for a correct linewidth of 1.0 mT, the red spectra for a too small linewidth of 0.2 mT and the blue spectra for a too large linewidth of 2.0 mT.

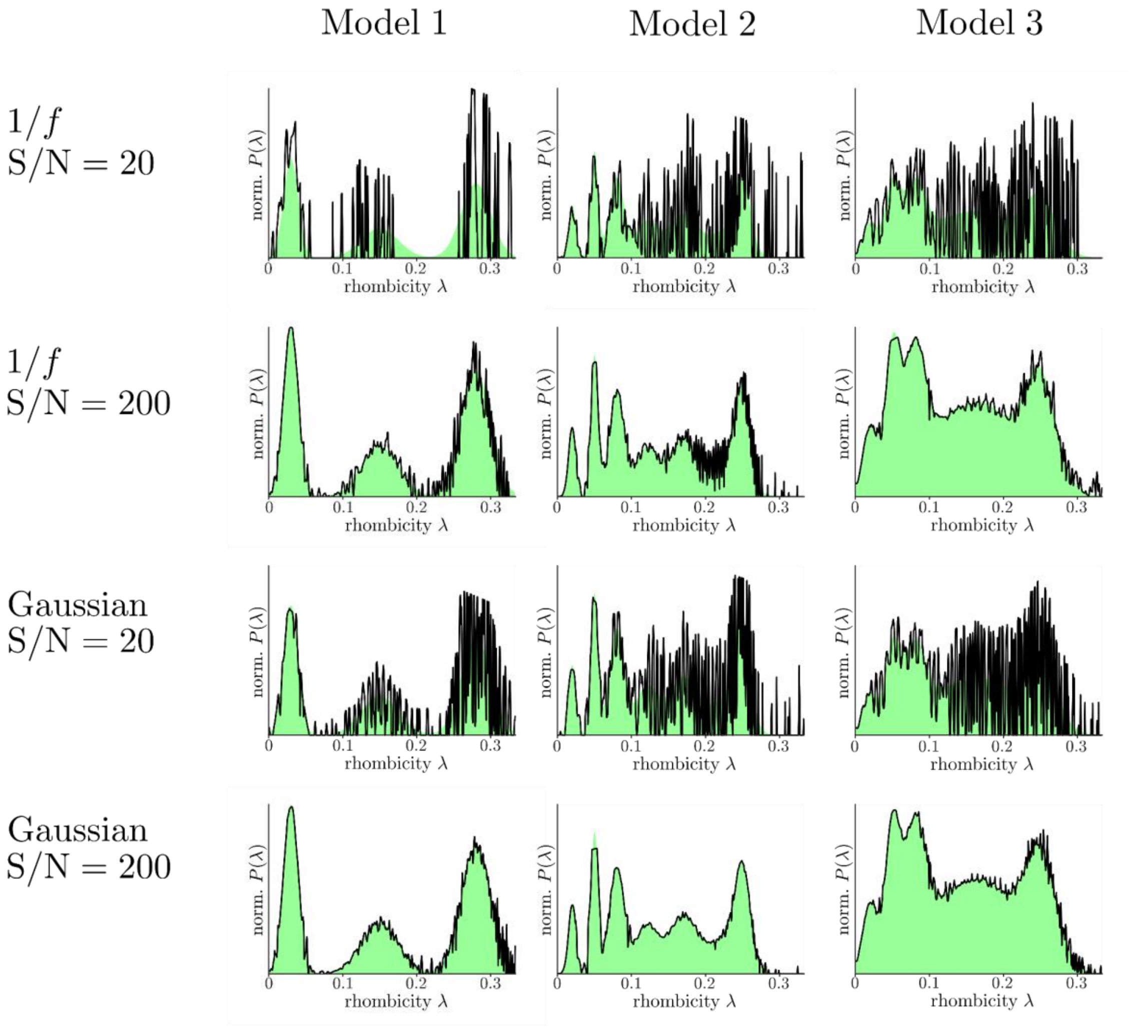

Supplementary Figure 13. Results of the Grid-of-Error method for models 1, 2 and 3 with different noise models and S/N ratios. The theoretical distribution functions $P_{\mathrm{Theo}}(\lambda)$ are shown as green areas, the black curves are the optimal results of the grid-of-error method.

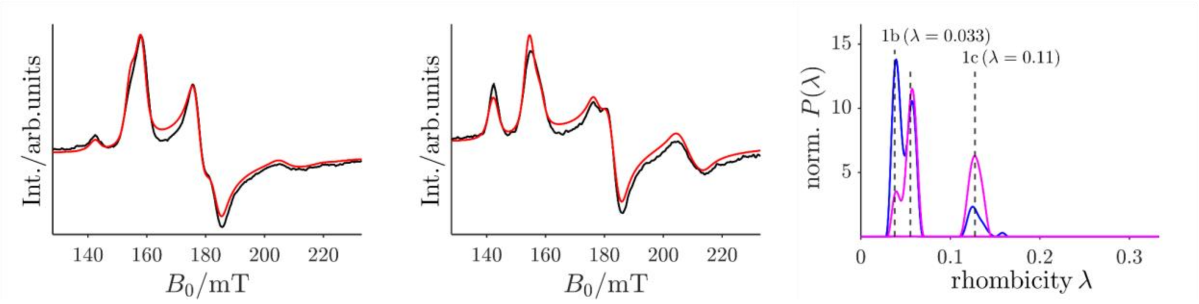

Supplementary Figure 14. Experimental cw-EPR spectra (black) [2] of freeze-quenched *Azotobacter vinelandii* nitrogenase complexes during turnover before (left) and after (middle) low-temperature blue light irradiation. Results from regularization are shown in red. Right: Corresponding probability distributions obtained from regularization before (blue) and after blue light illumination (magenta). The dashed lines are the λ-values which were computed from the effective g-values given by Lukoyanov et. al. [2]. The data sets were kindly provided by Prof. Brian Hoffman. A baseline correction and a data point interpolation was applied to reduce regularization artifacts.

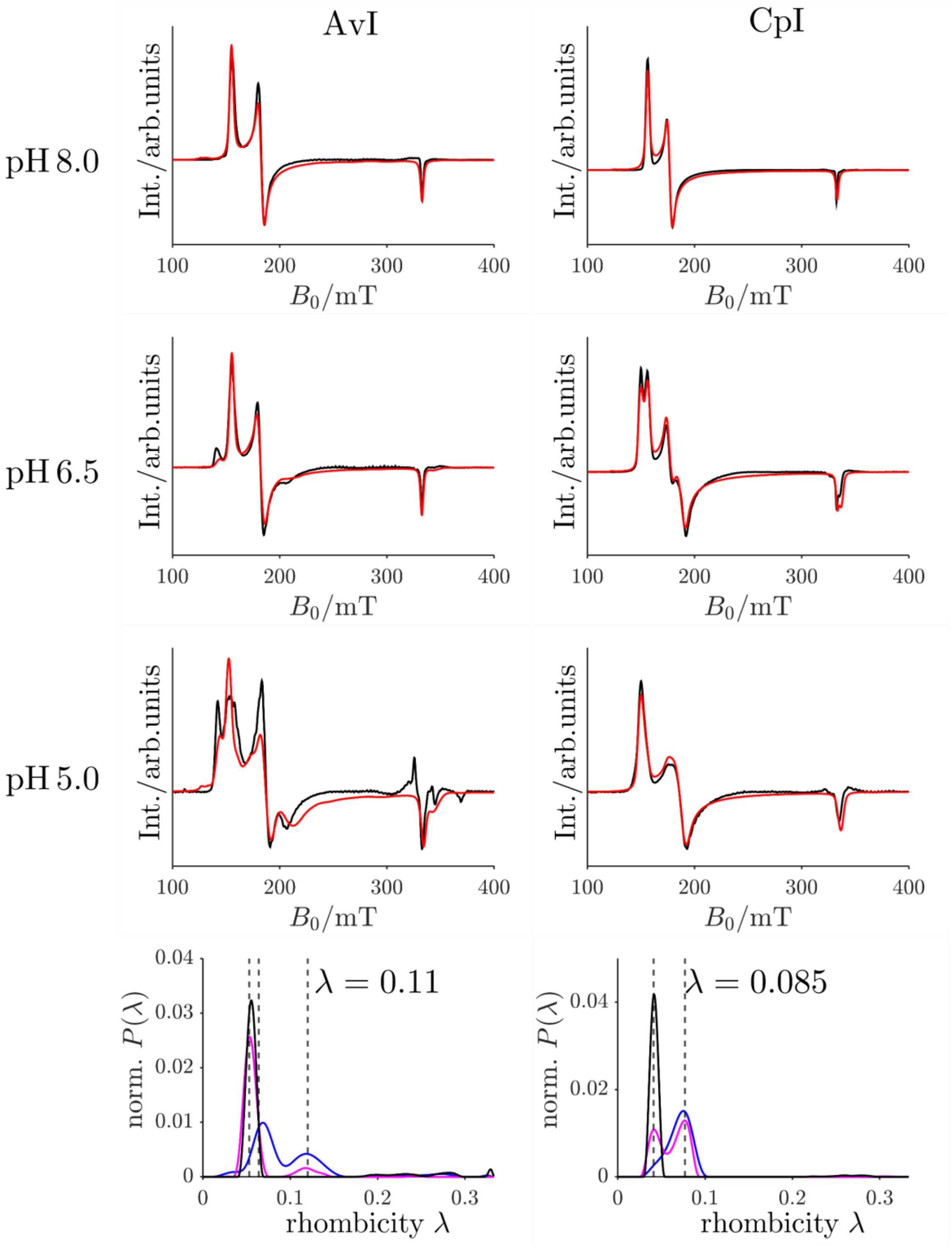

Supplementary Figure 15. Experimental cw-EPR spectra (black) of nitrogenase from *Azotobacter vinelandii* (AvI, left) and from *Clostridium pasteurianum* (CpI, right), recorded at three different pH values. Data were taken from [3], the results from regularization are depicted in red. Fourth row: The probability distributions obtained by the regularization at different pH values (black: pH 8.0, magenta: pH 6.5 and blue: pH 5.0). Dashed lines represent the published λ-values [3]. A baseline correction was applied to all data sets to reduce regularization artefacts.

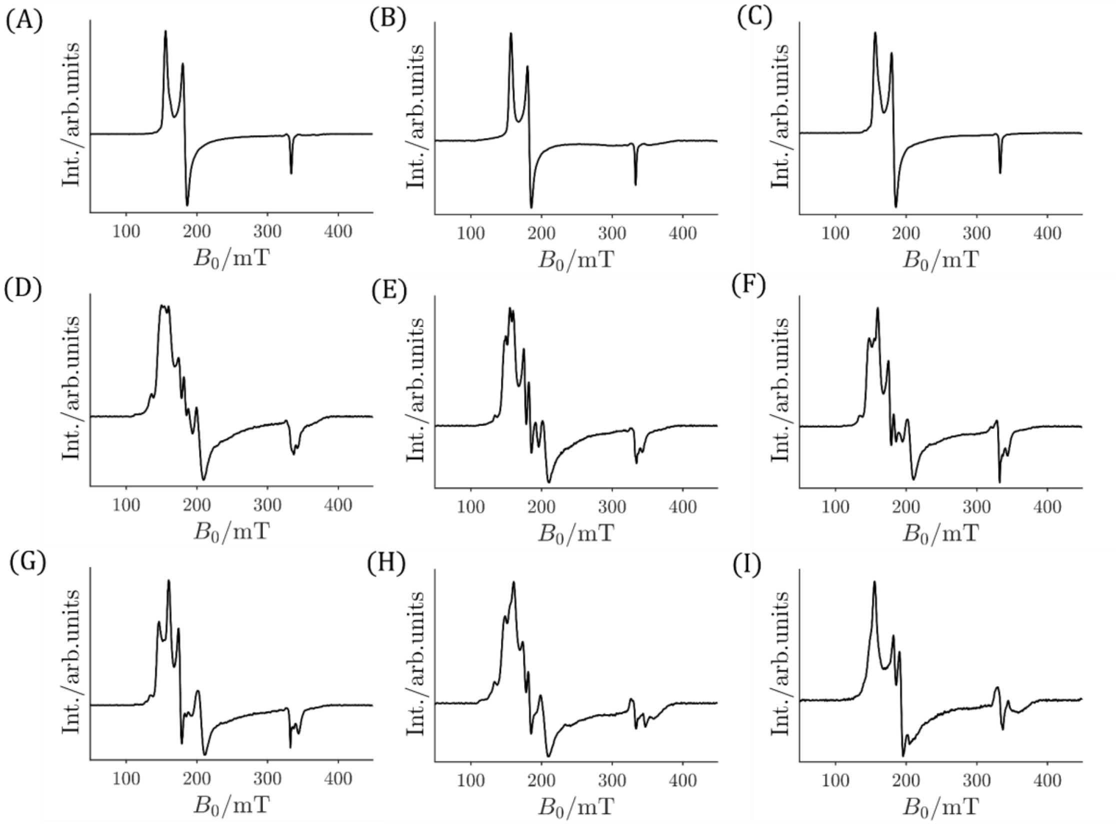

Supplementary Figure 16. Full-range X-Band cw-EPR spectra of samples (A) AvI-WT, (B) AvI-S, (C) AvI-^{33}S, (D) AvI-Se2B-1, (E) AvI-Se2B-lowflux, (F) AvI-Se-C_2H_2, (G) AvI-Se-low, (H) AvI-^{77}Se2B, (I) AvI-S-remigration. Spectra were measured with a microwave power of 37.7 mW at T = 5 K. Experimental details and parameters can be found in the Experimental section of the main text.

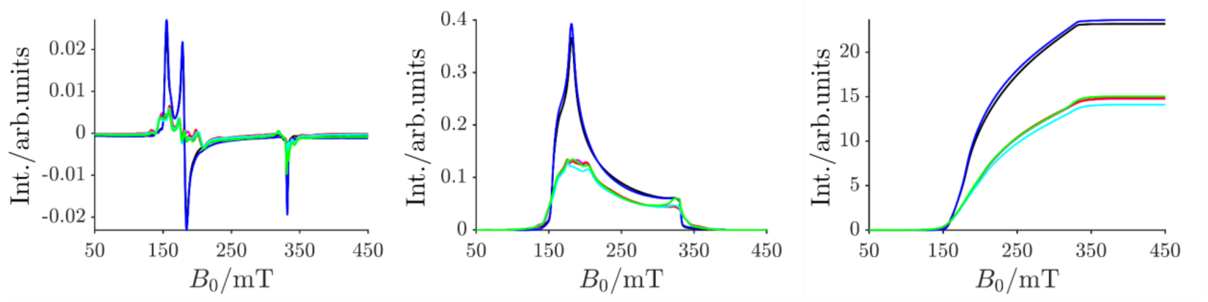

Supplementary Figure 17. Intensity comparison of the cw-EPR spectra of the differently prepared FeMo cofactors. To eliminate effects of the individual samples, all spectra were normalized to their respective protein concentration as well as to their sample volume, and a baseline was subtracted before and after each integration step while keeping all other parameters identical. The respective cw-EPR spectra are shown on the left, the integrated spectra in the center and the doubly integrated spectra are shown on the right side. This double integral corresponds to the total spin concentration of the respective spectrum. The individual samples are as follows: AvI-WT (black), AvI-Se2B-1 (magneta), AvI-^{77}Se (red), AvI-^{33}S (blue), AvI-Se-low (cyan) and AvI-Se-C_2H_2 (green). Experimental details and parameters can be found in the Experimental section of the main text.

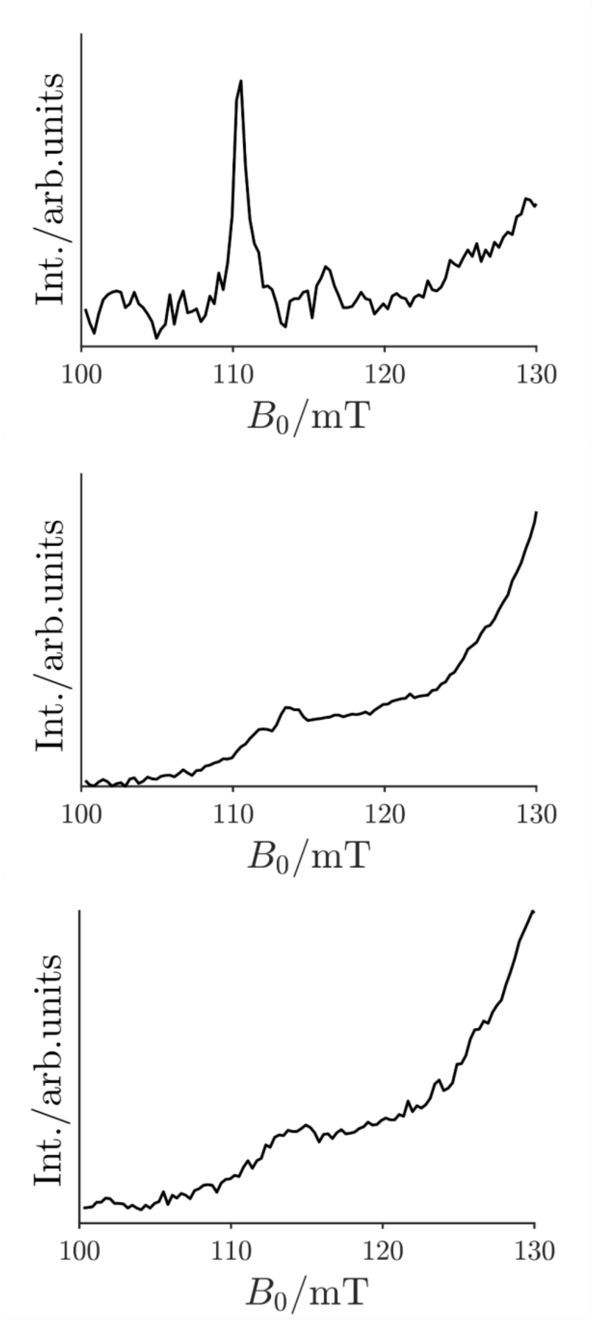

Supplementary Figure 18. X-band cw-EPR spectra of samples AvI-WT and AvI-Se2B-1 in the range of the resonances of the upper Kramers Doublet ($g_z^{3/2} \approx 6 \ = \approx 110$ mT). In the top panel the sample AvI-WT is shown at 6 K, and in the other two panels the sample AvI-Se2B-1 is shown at 6 K and at 15 K, respectively. Experimental details and parameters can be found in the Experimental section of the main text.

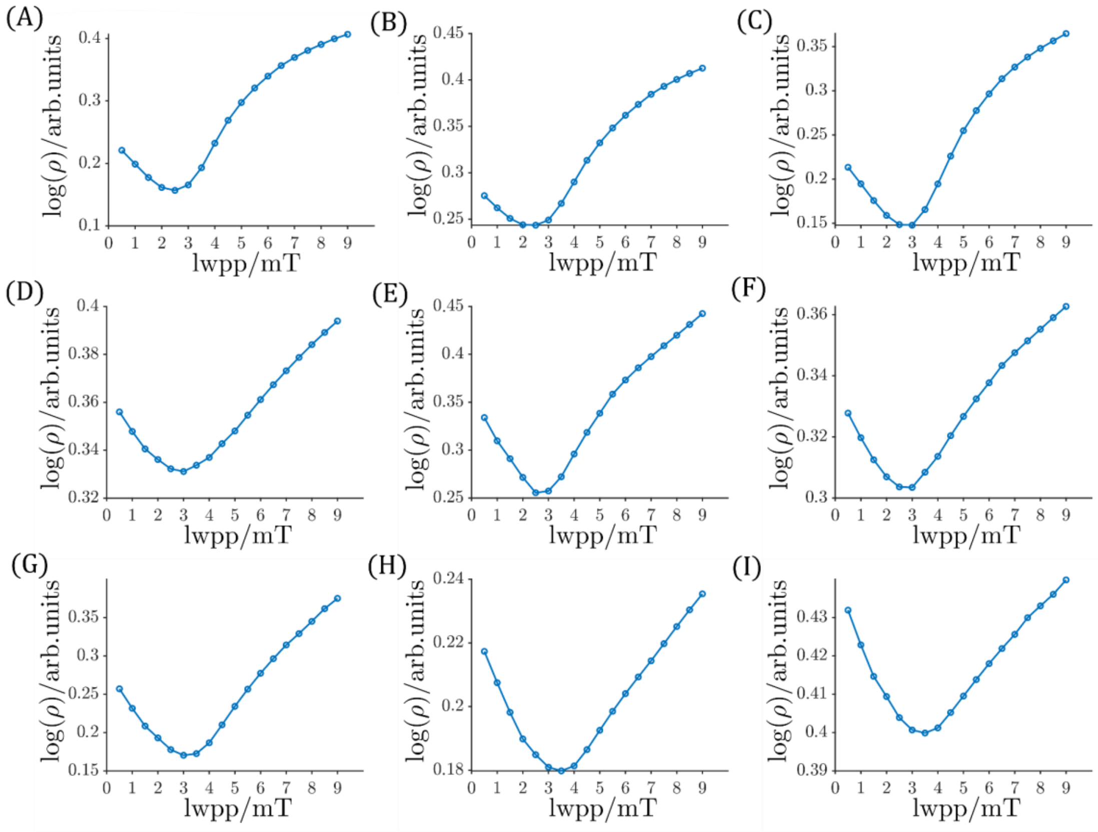

Supplementary Figure 19. Exemplary ρ(lwpp) graphs of samples (A) AvI-WT, (B) AvI-S, (C) AvI-^{33}S, (D) AvI-Se2B-1, (E) AvI-Se2B-lowflux, (F) AvI-Se-C_2H_2, (G) AvI-Se-low, (H) AvI-^{77}Se2B, (I) AvI-S-remigration, calculated from cw-EPR spectra measured with a microwave power of 0.095 mW at T = 6 K. Experimental details and parameters can be found in the Experimental section of the main text.

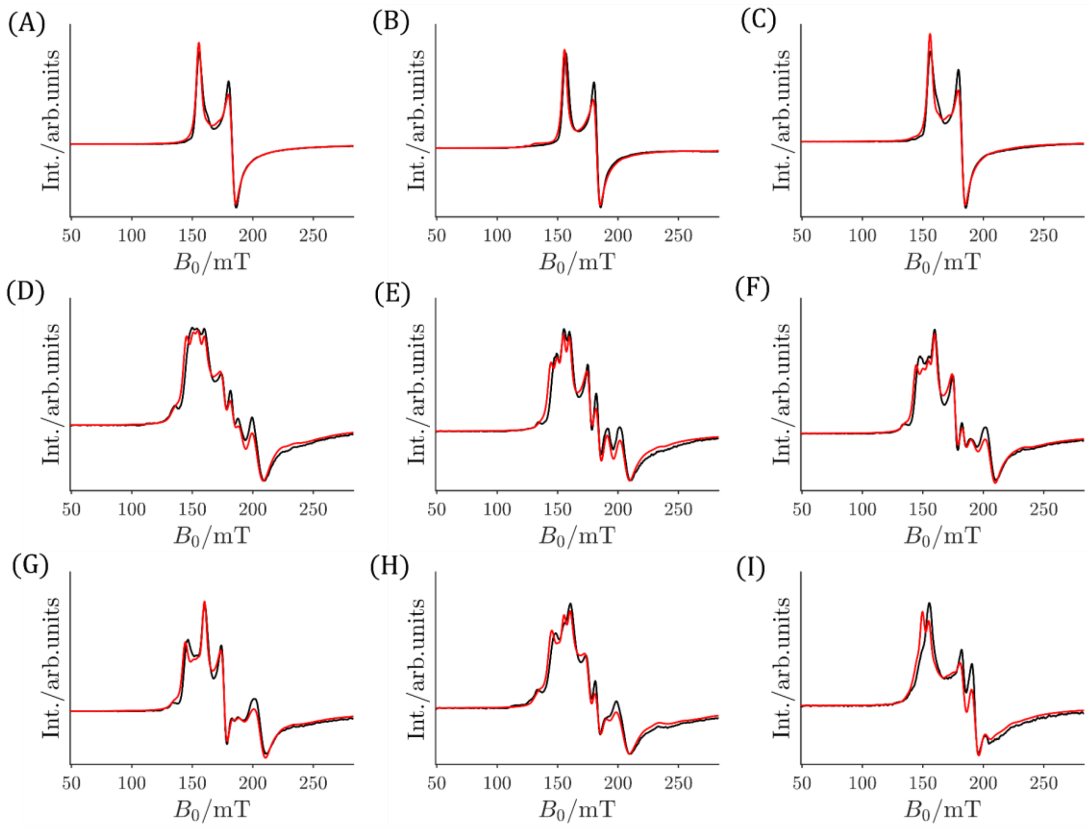

Supplementary Figure 20. X-Band cw-EPR spectra (black) of samples (A) AvI-WT, (B) AvI-S, (C) AvI-^{33}S, (D) AvI-Se2B-1, (E) AvI-Se2B-lowflux, (F) AvI-Se-C_2H_2, (G) AvI-Se-low, (H) AvI-^{77}Se2B, (I) AvI-S-remigration, measured with a microwave power of 0.095 mW at T = 6 K. Calculated spectra obtained from regularization using a linewidth of 2.5 mT are depicted in red. Experimental details and parameters can be found in the Experimental section of the main text.

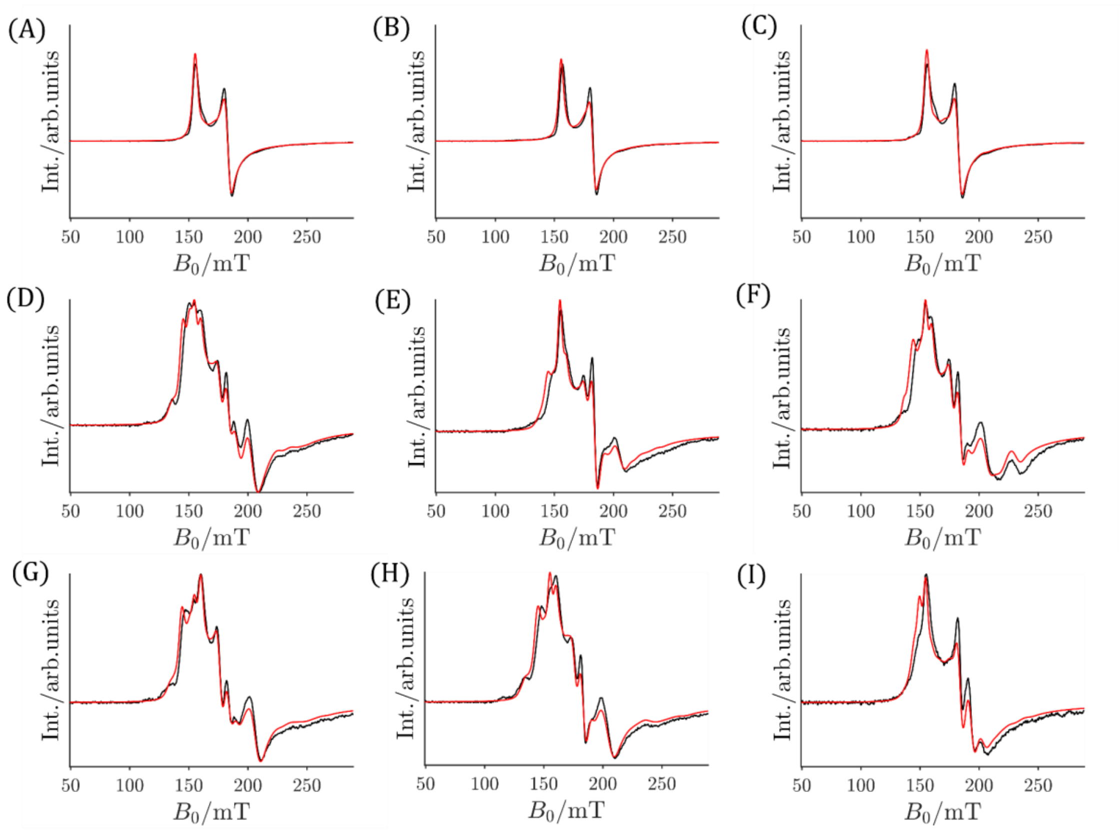

Supplementary Figure 21. X-Band cw-EPR spectra (black) of samples (A) AvI-WT, (B) AvI-S, (C) AvI-^{33}S, (D) AvI-Se2B-1, (E) AvI-Se2B-lowflux, (F) AvI-Se-C_2H_2, (G) AvI-Se-low, (H) AvI-^{77}Se2B, (I) AvI-S-remigration, measured with a microwave power of 0.095 mW at T = 9 K. Calculated spectra obtained from regularization using a linewidth of 3 mT are depicted in red. Experimental details and parameters can be found in the Experimental section of the main text.

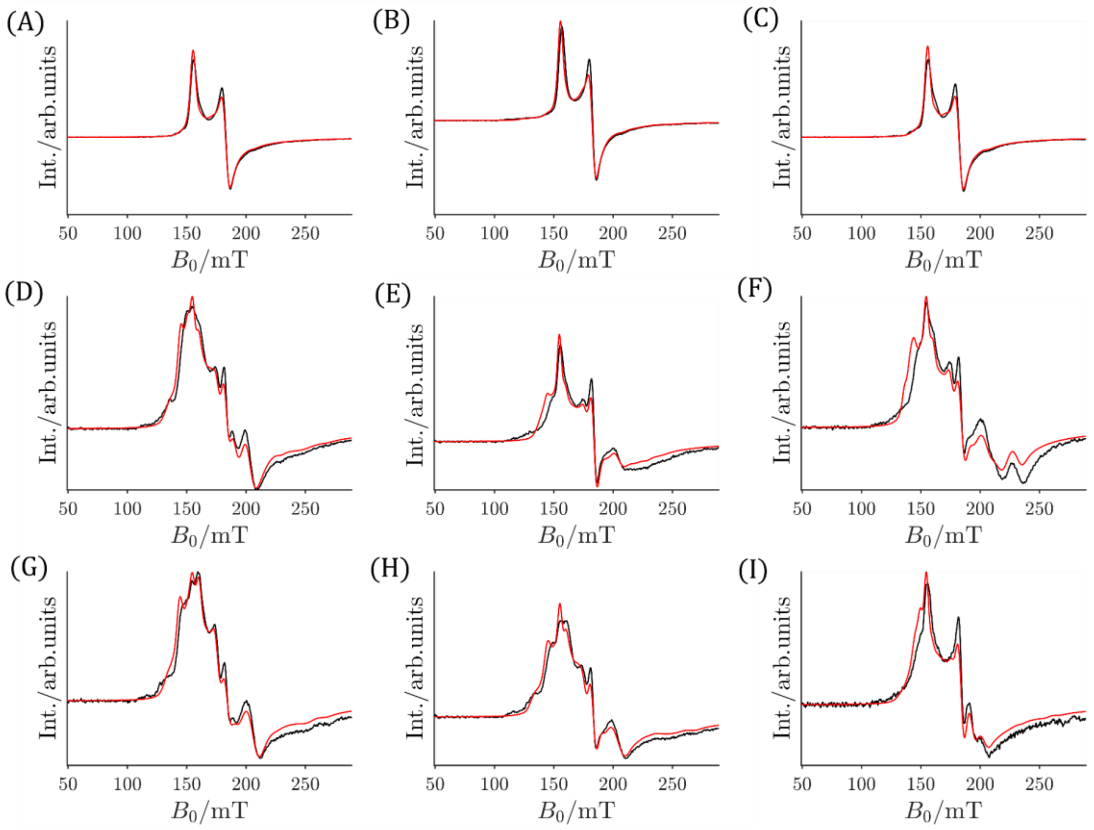

Supplementary Figure 22. X-Band cw-EPR spectra (black) of samples (A) AvI-WT, (B) AvI-S, (C) AvI-^{33}S, (D) AvI-Se2B-1, (E) AvI-Se2B-lowflux, (F) AvI-Se-C_2H_2, (G) AvI-Se-low, (H) AvI-^{77}Se2B, (I) AvI-S-remigration, measured with a microwave power of 0.095 mW at T = 12 K. Calculated spectra obtained from regularization using a linewidth of 3.5 mT are depicted in red. Experimental details and parameters can be found in the Experimental section of the main text.

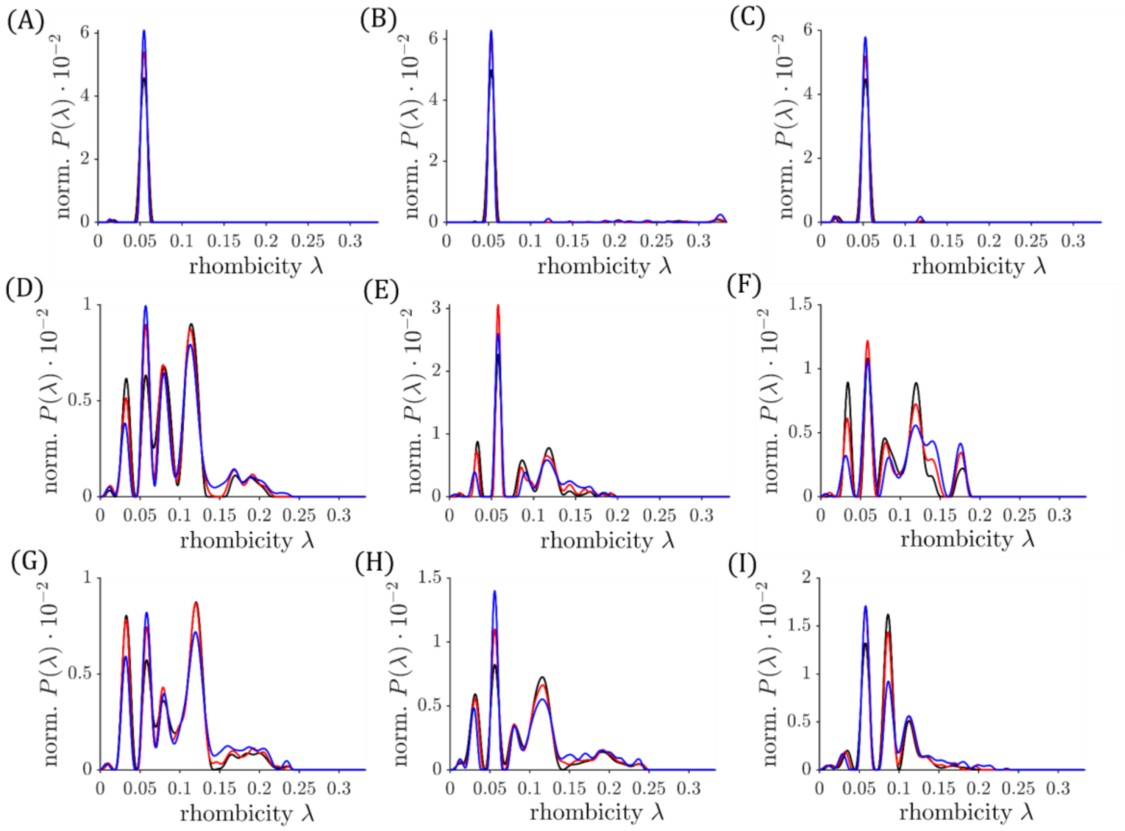

Supplementary Figure 23. Normalized probability distributions obtained from regularization of all cw-EPR spectra at 6 K (black), 9 K (red) and 12 K (blue) using a microwave power of 0.095 mW. Linewidths of 2.5, 3.0 and 3.5 mT were used for temperatures 6, 9 and 12 K, respectively. The samples are as follows: (A) AvI-WT, (B) AvI-S, (C) AvI-^{33}S, (D) AvI-Se2B-1, (E) AvI-Se2B-lowflux, (F) AvI-Se-C_2H_2, (G) AvI-Se-low, (H) AvI-^{77}Se2B, (I) AvI-S-remigration. Experimental details and parameters can be found in the Experimental section of the main text.

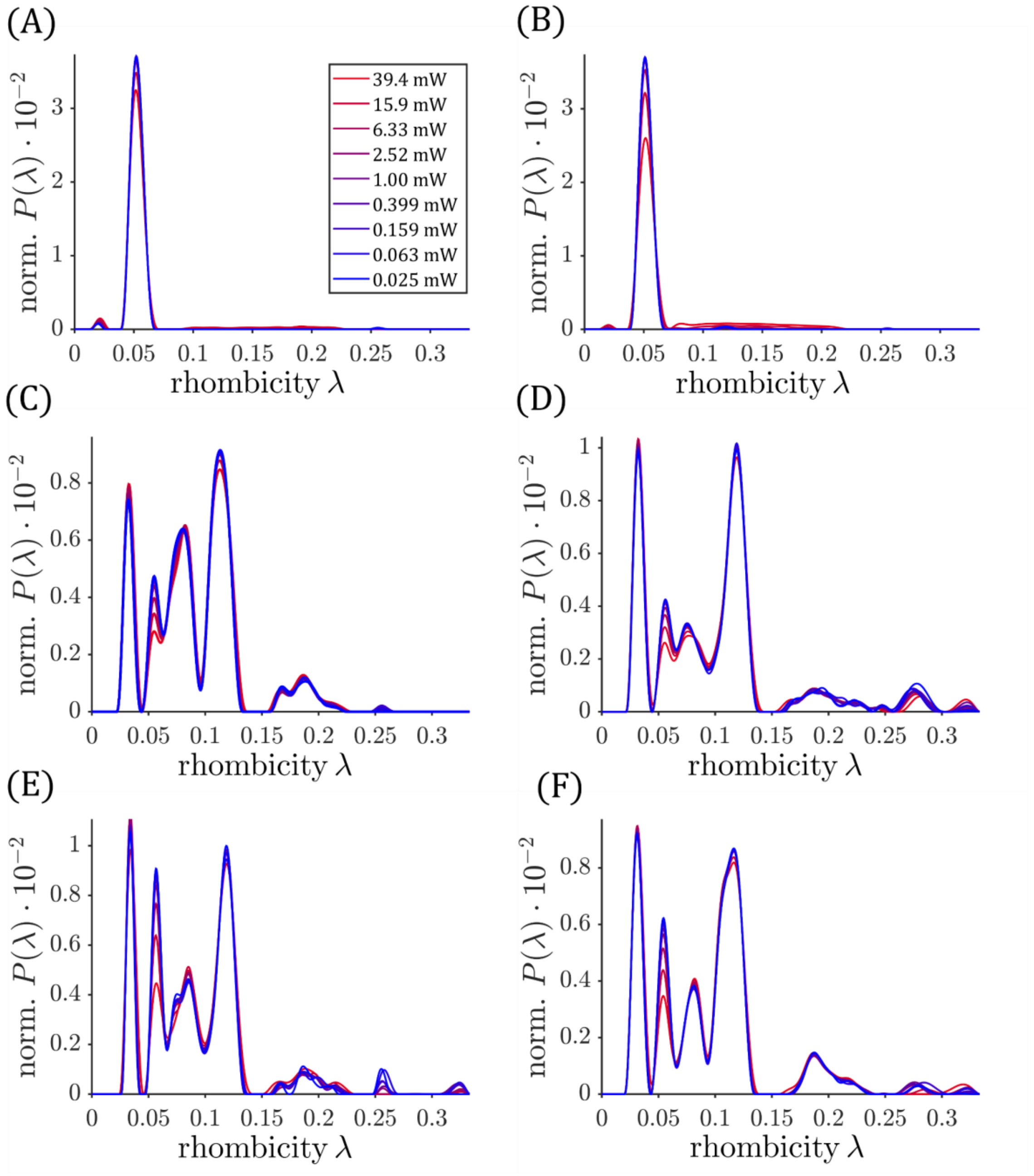

Supplementary Figure 24. Normalized probability distributions obtained from regularization of cw-EPR spectra at 5 K using different microwave powers (from 0.025 to 39.4 mW). An intrinsic linewidth of 2.0 mT was used for the regularization. The samples are as follows: (A) AvI-WT, (B) AvI-^{33}S; (C) AvI-Se2B-1; (D) AvI-Se-low; (E) AvI-Se-C_2H_2; (F) AvI-Se-^{77}Se. Experimental parameters: Modulation amplitude 0.6 mT, microwave frequency 9.37 GHz, conversion time 163.84 ms, time constant 335.54 ms.

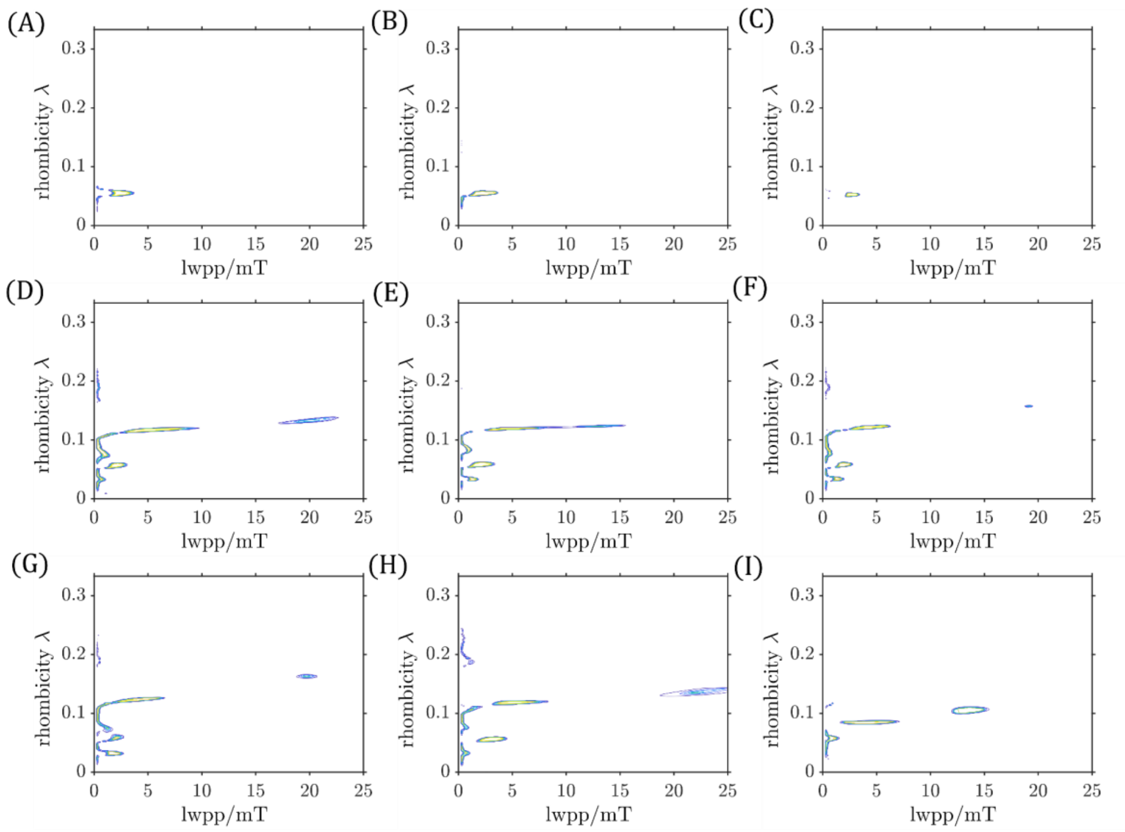

Supplementary Figure 25. 2-dimensional representation of the probability distribution $P(\lambda,\text{lwpp})$ obtained by the grid-of-errors method at a temperature of $T = 5$ K and a microwave power of 0.377 mW. The intensity of the distribution is shown in shades of color (from blue via green to yellow). The samples are as follows: (A) AvI-WT, (B) AvI-S, (C) AvI-^{33}S, (D) AvI-Se2B-1, (E) AvI-Se2B-lowflux, (F) AvI-Se-C_2H_2, (G) AvI-Se-low, (H) AvI-^{77}Se2B, (I) AvI-S-remigration. Experimental details and parameters can be found in the Experimental section of the main text.

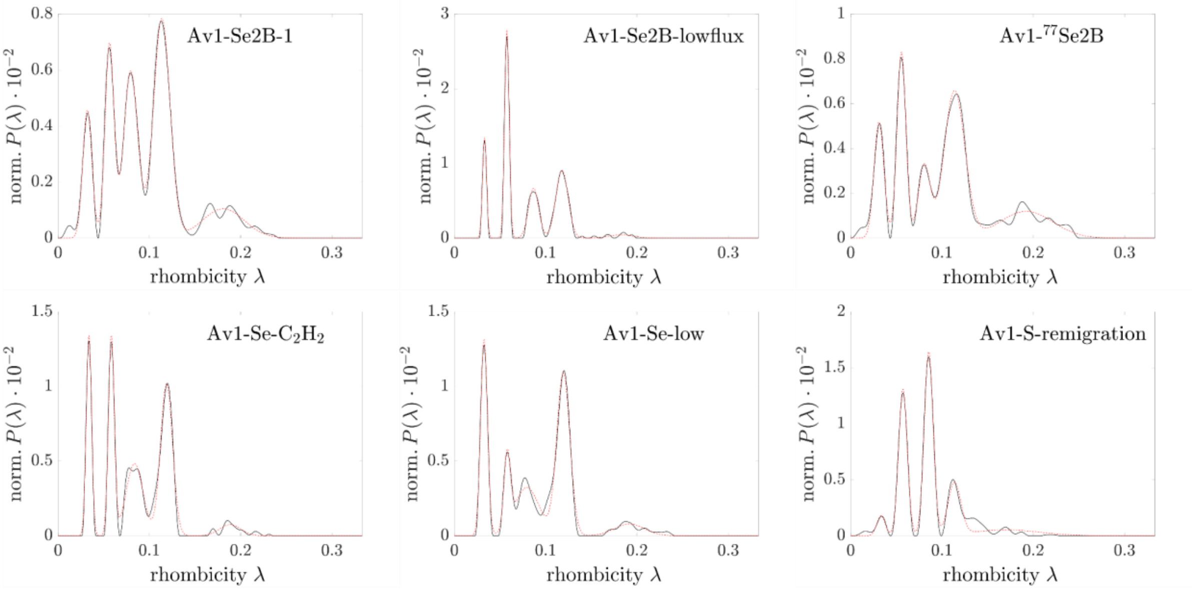

Supplementary Figure 26. Quantification of individual Se-species with a Multi-Gaussian fit. Regularizations taken from Figure 3 (black) of samples AvI-Se2B-1, Av1-Se2B-lowflux, AvI-^{77}Se2B, AvI-Se-C_2H_2, AvI-Se-low, and AvI-S-remigration were analyzed using up to five Gaussian functions (red dashed lines).

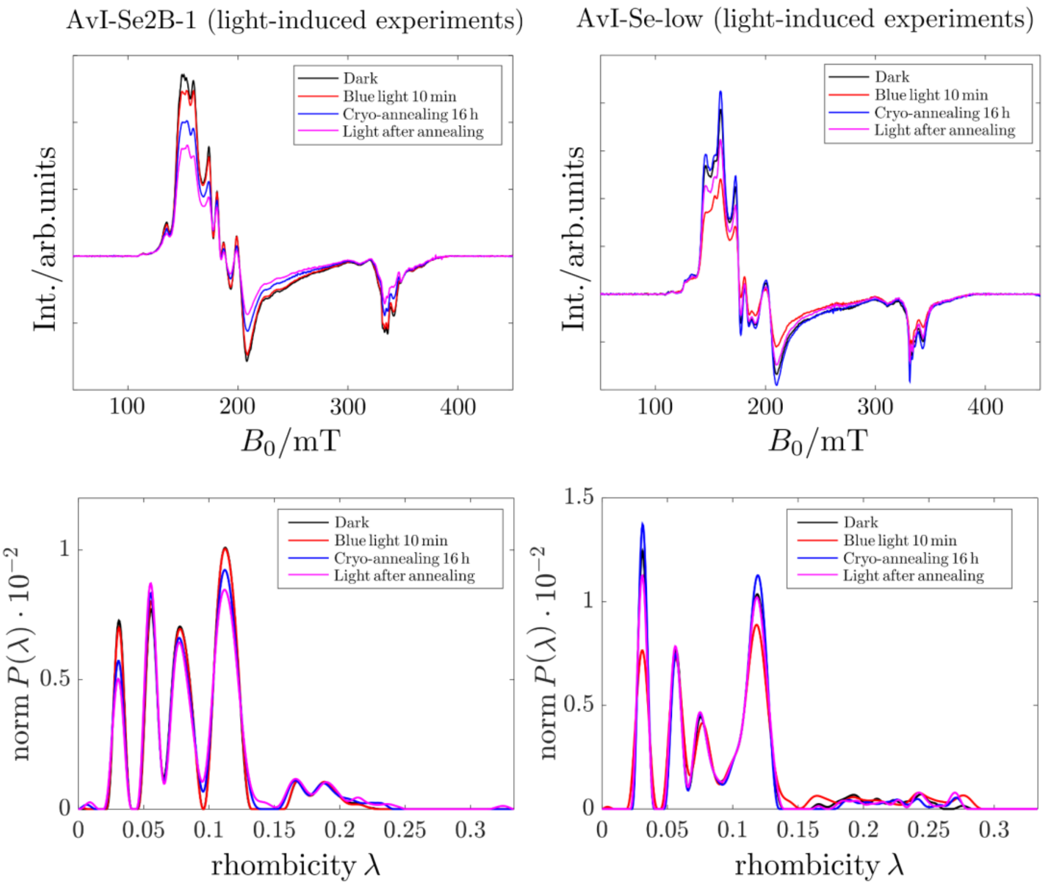

Supplementary Figure 27. Light-induced experiments of samples AvI-Se2B-1 (left) and AvI-Se-low (right). Samples were recorded in the dark (black), after 10 min of blue light illumination (red), after 16 h of cryo-annealing (blue), and after a second round of blue light illumination subsequent to cryo-annealing (magenta). Cw-EPR spectra are shown in the upper panels, normalized distributions obtained by regularization are shown in the lower panels. For experimental details, see Methods section.

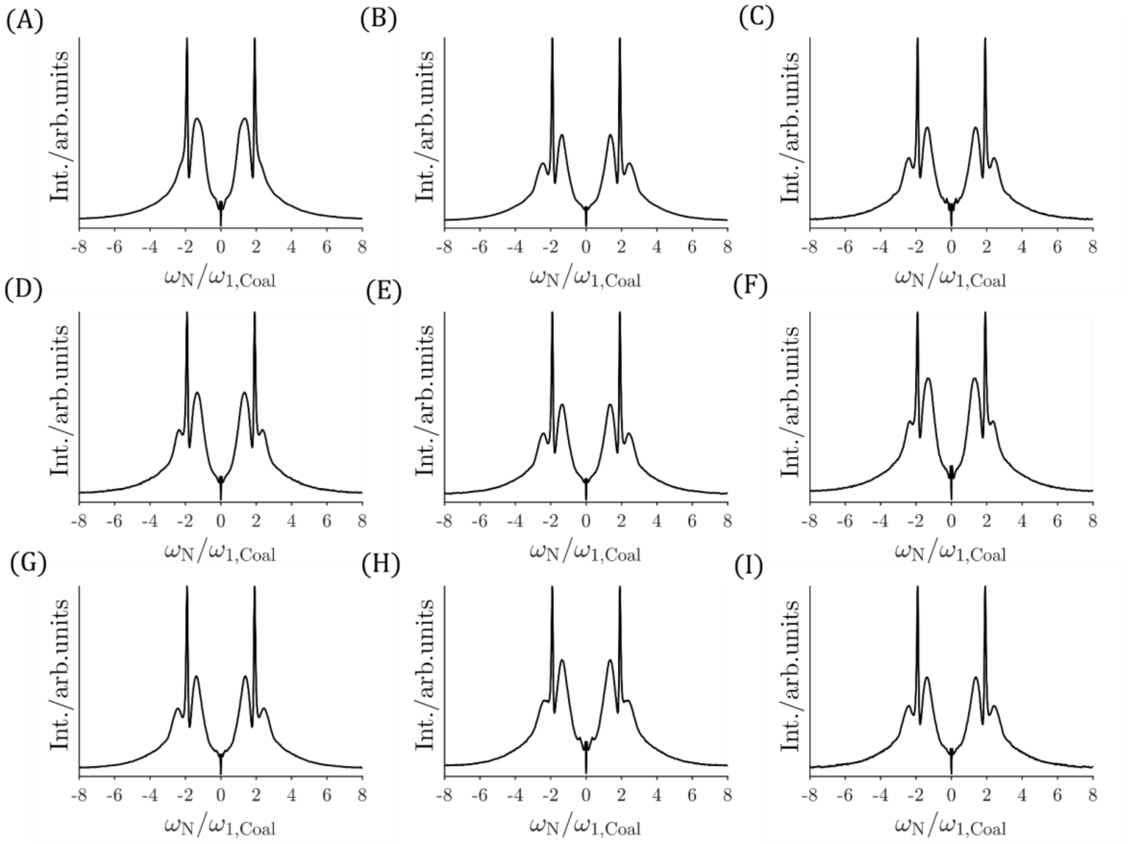

Supplementary Figure 28. Transient nutation experiments at B_0 = 660 mT using a microwave frequency of 33.8 GHz. The samples are depicted as follows: (A) AvI-WT, (B) AvI-S, (C) AvI-^{33}S, (D) AvI-Se2B-1, (E) AvI-Se2B-lowflux, (F) AvI-Se-C_2H_2, (G) AvI-Se-low, (H) AvI-^{77}Se2B, (I) AvI-S-remigration. Experimental details and parameters can be found in the Experimental section of the main text.

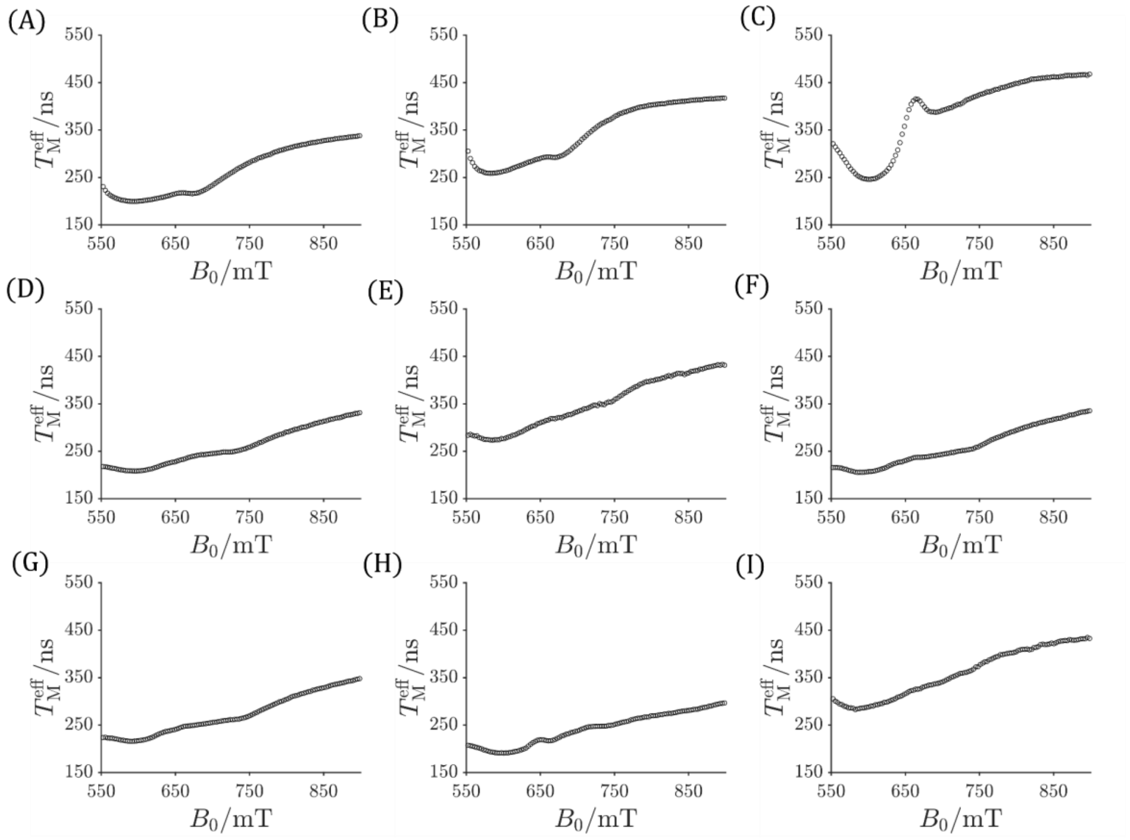

Supplementary Figure 29. Magnetic field dependence of T_M^{eff} for samples (A) AvI-WT, (B) AvI-S, (C) AvI-^{33}S, (D) AvI-Se2B-1, (E) AvI-Se2B-lowflux, (F) AvI-Se-C_2H_2, (G) AvI-Se-low, (H) AvI-^{77}Se2B, (I) AvI-S-remigration. Experimental details and parameters can be found in the Experimental section of the main text.

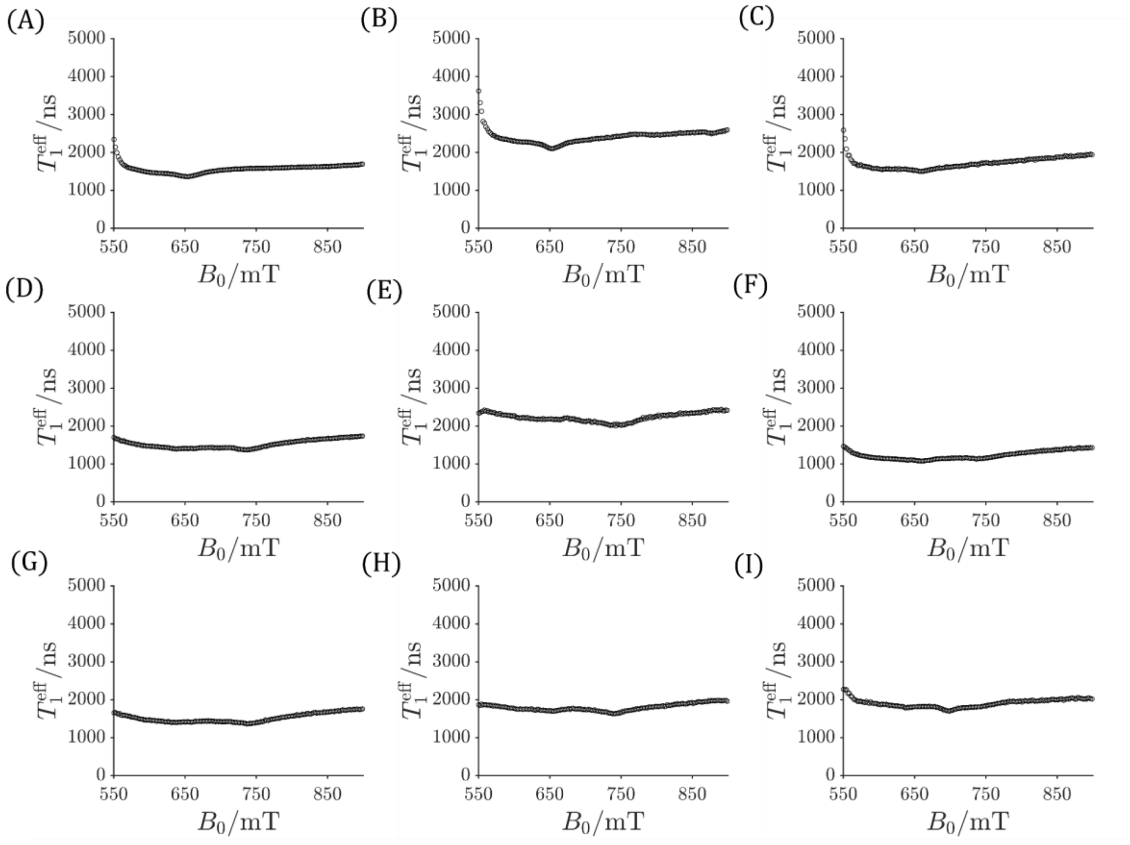

Supplementary Figure 30. Magnetic field dependence of T_1^{eff} for samples (A) AvI-WT, (B) AvI-S, (C) AvI-^{33}S, (D) AvI-Se2B-1, (E) AvI-Se2B-lowflux, (F) AvI-Se-C_2H_2, (G) AvI-Se-low, (H) AvI-^{77}Se2B, (I) AvI-S-remigration. Experimental details and parameters can be found in the Experimental section of the main text.

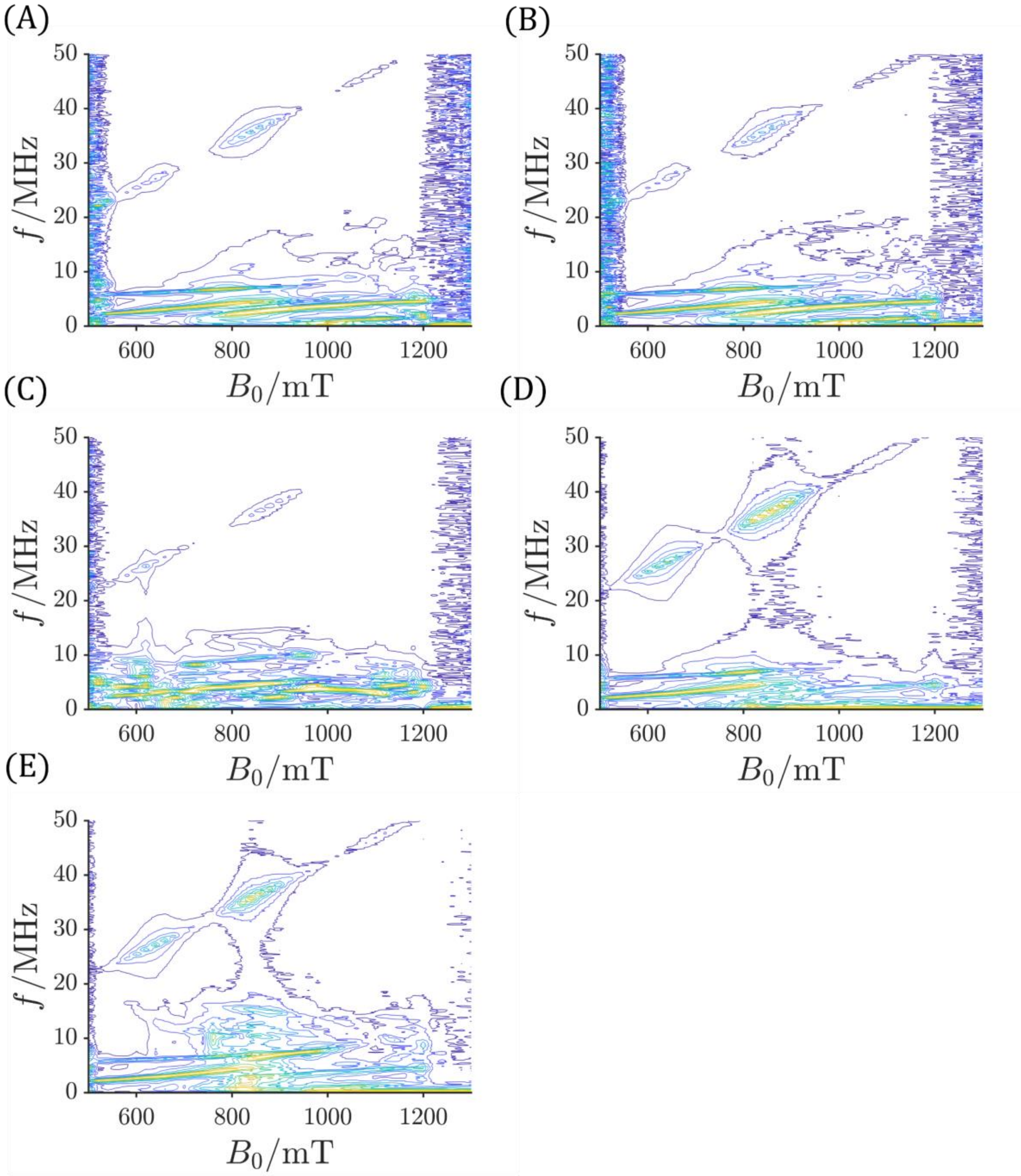

Supplementary Figure 31. Magnetic-field dependent 3P-ESEEM experiments recorded at Q-band (microwave frequency 33.8 GHz). The samples are shown as follows: (A) AvI-WT, (B) AvI-S, (C) AvI-^{33}S, (D) AvI-Se2B-1, (E) AvI-^{77}Se2B. The intensities are depicted as a color gradient from low (blue) via green to high intensity (yellow/orange). In order to better visualize the intensities and couplings, each 3P-ESEEM spectrum was normalized to the respective maximum for each magnetic field point.

Supplementary Tables:

Supplementary Table 1. λ-mean values, standard deviations σ_i, and weighting w_i of each Gaussian distribution of the three model systems. The theoretical distribution P_{Theo} is then given by:

$$P_{\text{Theo}}(\lambda) = \sum_i w_i g_i(\lambda) \qquad \text{(Eq. 1)}$$

with

$$g_i(\lambda) = \frac{1}{\sqrt{2\pi\sigma_i^2}} \exp\left(-\frac{(\lambda-\bar{\lambda}_i)^2}{2\sigma_i^2}\right) \qquad \text{(Eq. 2)}$$

model 1			model 2			model 3		
λ-mean value $\bar{\lambda}_i$	standard deviation σ_i	statistical weight w_i	λ-mean value $\bar{\lambda}_i$	standard deviation σ_i	statistical weight w_i	λ-mean value $\bar{\lambda}_i$	standard deviation σ_i	statistical weight w_i
0.03	0.010	0.4	0.02	0.005	0.20	0.02	0.010	0.20
0.15	0.025	0.3	0.05	0.005	0.40	0.05	0.010	0.40
0.28	0.020	0.6	0.08	0.010	0.60	0.08	0.015	0.60
			0.12	0.015	0.40	0.12	0.025	0.40
			0.17	0.020	0.65	0.17	0.030	0.65
			0.23	0.022	0.45	0.23	0.035	0.45
			0.25	0.010	0.50	0.25	0.020	0.50

Supplementary Table 2. Multi-Gaussian Fitting of the probability distributions of Se-modified samples using a fit with 5-Gaussian functions. The statistical weights g_k are listed on the right side of the Table.

	$\boldsymbol{\lambda_k}$- from fit					$\boldsymbol{w_k}$- from fit: $\times\ \mathbf{10^{-4}}$					
Sample	$\boldsymbol{\lambda_1}$	$\boldsymbol{\lambda_2}$	$\boldsymbol{\lambda_3}$	$\boldsymbol{\lambda_4}$	$\boldsymbol{\lambda_5}$	$\boldsymbol{w_1}$	$\boldsymbol{w_2}$	$\boldsymbol{w_3}$	$\boldsymbol{w_4}$	$\boldsymbol{w_5}$	$\boldsymbol{w_{\text{tot}}}$
Av1-Se2B-1	0.032	0.056	0.078	0.114	0.181	0.572	0.854	1.159	1.784	0.599	4.968
Av1-Se2B-lowflux	0.033	0.058	0.087	0.112	0.183	0.736	1.745	0.935	1.531	0.148	5.094
Av1-^{77}Se2B	0.031	0.056	0.081	0.114	0.192	0.700	1.004	0.527	1.870	0.871	4.972
Av1-Se-C_2H_2	0.034	0.059	0.084	0.119	0.188	0.969	1.053	1.126	1.691	0.219	5.058
Av1-Se-low	0.033	0.058	0.079	0.119	0.191	1.223	0.424	1.110	1.963	0.327	5.048
Av1-S-remigration	0.033	0.057	0.085	0.112	0.137	0.205	1.541	2.001	0.601	0.609	4.958

Supplementary Notes:

Theoretical background of the regularization and grid-of-errors methods.

Protein-cofactor interactions modulate selectively the properties of (metal) cofactors, leading to an inhomogeneous broadening of their magnetic parameters. Such effects can be approximated by a random distribution of the EPR parameters, especially the $\boldsymbol{D}$-tensor and the $\boldsymbol{g}$-tensor, simply by using Gaussian distributions, so-called strain models[4-7]. These simple strain models are valid as long as the width of the distribution is small compared to its magnitude.

On the other hand, zero-field splitting strains on high-spin systems are often large with respect to their magnitude and hence, their correct description is crucial for a correct analysis of EPR data sets[8]. If the distribution of an EPR relevant parameter is broad and/or of unknown distribution, strain models cannot reproduce experimental datasets correctly and hence, other approaches become necessary. In this study, the EPR spectra of various Se- and S-exchanged FeMo cofactors were analyzed by two independent methods: A Tikhonov regularization and the grid-of-error approach[8] were utilized to analyze the complicated splitting patterns and the result compared in terms of accuracy and speed. Grid methods can be very time-consuming if more than one parameter is modelled, but they are highly potent for obtaining unknown parameter distributions. Both methods are introduced in the next paragraph.

Spin-Hamiltonian of the FeMo-Cofactor.

The EPR spectra of a high-spin system ($S > 1/2$) can be described by the following Hamiltonian: $H = H_{\mathrm{Zee}} + H_{\mathrm{ZFS}} = \mu_{\mathrm{B}}\boldsymbol{SgB_0} + \boldsymbol{SDS}$. Here, H_{Zee} is the electron Zeeman Hamiltonian and $H_{\mathrm{ZFS}} = D\left(S_{\mathrm{z}}^2 - \frac{S(S+1)}{3}\right) + E(S_{\mathrm{x}}^2 - S_{\mathrm{y}}^2) = D\left(S_{\mathrm{z}}^2 - \frac{S(S+1)}{3} \pm \lambda(S_{\mathrm{x}}^2 - S_{\mathrm{y}}^2)\right)$ describes the zero-field-Hamiltonian with D as the zero-field splitting parameter and $\lambda = |E/D|$ as the rhombicity $\left(0 \leq \lambda \leq \frac{1}{3}\right)$[9]. The Hamiltonian of a zero-field splitting together with the electronic Zeeman Hamiltonian can be solved for the magnetic field along the principal $\boldsymbol{g}$-tensor axes[10]. For the resting state of FeMo cofactor in Av1, typical zero-field splitting parameters are $D \approx 180$ GHz, $\lambda \approx 0.05$ and a total spin quantum number of S = 3/2. This leads to a splitting of the energy levels $\left(m_s = \pm\frac{1}{2} \text{ and } m_s = \pm\frac{3}{2}\right)$ of $\Delta = 2D\sqrt{1+3\lambda^2} \approx 360$ GHz[11,12]. (For small λ-values, $\Delta \approx 2D$ can be assumed). As the electron Larmor frequency, defined by the external magnetic field B_0, is much smaller than the zero-field splitting, $|\Delta| \gg |\mu_B B_0 g| = f_{\mathrm{MW}}$, both Kramers doublets can be treated separately and each doublet can be described by a fictitious $S' = 1/2$ spin system with effective g'-tensor components. In this case the effective principal g'-values can be determined from the energy levels of the separated Kramers duplets[10,13,14]:

$$
\begin{aligned}
g'^{1/2}_{\mathrm{z}} &= g_{\mathrm{z}}\left[\frac{2}{\sqrt{1+3\lambda^2}} - 1\right] & g'^{3/2}_{\mathrm{z}} &= g_{\mathrm{z}}\left[\frac{2}{\sqrt{1+3\lambda^2}} + 1\right] \\
g'^{1/2}_{\mathrm{y}} &= g_{\mathrm{y}}\left[\frac{1+3\lambda}{\sqrt{1+3\lambda^2}} + 1\right] & g'^{3/2}_{\mathrm{y}} &= g_{\mathrm{y}}\left[\frac{1+3\lambda}{\sqrt{1+3\lambda^2}} - 1\right] \qquad \text{(Eq. 3)} \\
g'^{1/2}_{\mathrm{x}} &= g_{\mathrm{x}}\left[\frac{1-3\lambda}{\sqrt{1+3\lambda^2}} + 1\right] & g'^{3/2}_{\mathrm{x}} &= g_{\mathrm{x}}\left[-\frac{1-3\lambda}{\sqrt{1+3\lambda^2}} + 1\right]
\end{aligned}
$$

The superscripts $1/2$ and $3/2$ denote the lower and upper Kramers doublet, respectively, and the subscript denotes the principal axis of the respective $\boldsymbol{g}$-tensor. The equations clearly show that the principal values of the $\boldsymbol{g}'$-tensor are functions of λ. In case of $\Delta > 0$, the Kramers doublet $m_S = \pm 1/2$ is lower in energy than the Kramers doublet $m_S = \pm 3/2$, and thus the lower Kramers doublet dominates the EPR spectrum of the FeMo cofactor in its resting-state. Please note that the Kramers doublet $m_S = \pm 1/2$ obeys the selection rule $\Delta m_S = \pm 1$, while the Kramers doublet $m_S = \pm 3/2$ obeys $\Delta m_S = \pm 3$ and thus is an unlikely transition.

Tikhonov regularization.

A data set of a cw-EPR spectrum, $y(B_0)$, is assumed. This data set with n data points can be described by a statistical probability distribution density $p(\lambda)$ and can be written as a Fredholm integral $y = y(B_0) = \int_{b_1}^{b_2} a(\lambda, B_0) p(\lambda) \mathrm{d}\lambda$ with $a(\lambda, B_0)$ being the kernel[15]. By discretization of the distribution $p(\lambda)$ in k steps, the integral can be approximated by $\boldsymbol{y} \approx \boldsymbol{A} \cdot \boldsymbol{P}$ with $\boldsymbol{A}$ being a $n \times k$ matrix and $\boldsymbol{P} = \boldsymbol{P}(\lambda)$ a vector of length k[16,17]. This matrix equation $\boldsymbol{y} = \boldsymbol{A} \cdot \boldsymbol{P}$ leads to an ill-posed problem, which can be solved by regularization[16,18-22]. In principle, such a problem is similar to the determination of probability distributions in PELDOR/DEER data sets, and therefore, Tikhonov regularization can be applied[15,23,24]. Its solution is[20-22]:

$$\widehat{\boldsymbol{P}}(\lambda) = \underset{P(\lambda)\geq 0}{\operatorname{argmin}} \left(\frac{1}{2} \|\boldsymbol{AP} - \boldsymbol{y}\|_2^2 + \frac{\alpha^2}{2} \|\boldsymbol{LP}\|_2^2 \right) \qquad \text{(Eq. 4)}$$

with $\|\ldots\|_2$ being the Euclidian metric and $\boldsymbol{L}$ the regularization operator. The constraint $\boldsymbol{P}(\lambda) \geq 0$ is necessary as $\boldsymbol{P}(\lambda)$ is a positive probability distribution. Usually, the identity operator ($\boldsymbol{L} = \boldsymbol{L}_0 = \mathbf{id}$), the first (discrete) derivative ($\boldsymbol{L} = \boldsymbol{L}_1$) or the second (discrete) derivative ($\boldsymbol{L} = \boldsymbol{L}_2$) are used[22]:

$$\boldsymbol{L}_0 = \begin{pmatrix} 1 & 0 & \ldots & 0 \\ 0 & \ddots & \ddots & \vdots \\ \vdots & \ddots & \ddots & 0 \\ 0 & \ldots & 0 & 1 \end{pmatrix}; \ \boldsymbol{L}_1 = \begin{pmatrix} -1 & 1 & & 0 \\ 0 & \ddots & \ddots & \\ & \ddots & \ddots & 1 \\ 0 & & 0 & -1 \end{pmatrix}; \boldsymbol{L}_2 = \begin{pmatrix} -2 & 1 & & 0 \\ 1 & \ddots & \ddots & \\ & \ddots & \ddots & 1 \\ 0 & & 1 & -2 \end{pmatrix} \text{(Eq. 5)}$$

The parameter α is the regularization parameter. Overly large values of α lead to over-smoothing of the results, while too small α values lead to artifacts due to ill-posed problems[16]. The determination of the optimal α value (α_{Opt}) is thus of high importance to find the best solution of $\widehat{\boldsymbol{P}}(\lambda)$. Several established methods were used to determine α_{Opt}[21,22]. First, the L-curve criterion[22,25] was used:

$$\alpha_{\mathrm{Opt}} = \underset{\alpha}{\operatorname{argmin}} \left\{ \left(\frac{\rho - \rho_{\min}}{\rho_{\max} - \rho_{\min}} \right)^2 + \left(\frac{\eta - \eta_{\min}}{\eta_{\max} - \eta_{\min}} \right)^2 \right\} \qquad \text{(Eq. 6)}$$

with $\rho = \log\left(\left\| \boldsymbol{A}\widehat{\boldsymbol{P}} - \boldsymbol{y} \right\|_2^2 \right)$ and $\eta = \log\left(\left\| \boldsymbol{L}\widehat{\boldsymbol{P}} \right\|_2^2 \right)$. Second, the generalized cross validation (GCV) method was used[22,26,27]:

$$\alpha_{\mathrm{Opt}} = \underset{\alpha}{\operatorname{argmin}} \left\{ \frac{\left\| \boldsymbol{A}\widehat{\boldsymbol{P}} - \boldsymbol{y} \right\|_2^2}{\left(1 - \frac{\mathrm{tr}(\boldsymbol{H}_\alpha)}{k} \right)^2} \right\} \qquad \text{(Eq. 7)}$$

with $\boldsymbol{H}_\alpha = \boldsymbol{A}\left(\boldsymbol{A}^\mathrm{T}\boldsymbol{A} + \alpha^2\boldsymbol{L}^\mathrm{T}\boldsymbol{L}\right)^{-1}A^\mathrm{T}$, tr(...) is the trace of the matrix and k as the rank of the matrix H_α, while $^\mathrm{T}$ denotes the transposed matrix. Moreover, the robust GCV method (rGCV) was tested[22,28]:

$$\alpha_\mathrm{Opt} = \underset{\alpha}{\operatorname{argmin}}\left\{\frac{\|\boldsymbol{A}\widehat{\boldsymbol{P}}-\boldsymbol{y}\|_2^2}{\left(1-\frac{\mathrm{tr}(\boldsymbol{H}_\alpha)}{k}\right)^2}\left[\gamma + (1-\gamma)\mathrm{tr}\left(\frac{\boldsymbol{H}_\alpha^2}{k}\right)\right]\right\} \qquad \text{(Eq. 8)}$$

with $\gamma = 0.8$. Finally, two information criterion techniques, the Akaike information criterion (AIC)[29] and the Bayesian information criterion (BIC)[30], were tested[21,22]:

$$\alpha_\mathrm{Opt} = \underset{\alpha}{\operatorname{argmin}}\left\{k\ln\left(\frac{\|\boldsymbol{A}\widehat{\boldsymbol{P}}-\boldsymbol{y}\|_2^2}{k}\right) + b\,\mathrm{tr}(\boldsymbol{H}_\alpha)\right\} \qquad \text{(Eq. 9)}$$

$b = 4$ was used for AIC and $b = 2\ln(k)$ for BIC.

To solve the ill-posed problem, Eq. 4 can be transformed into a quadric programming problem by using $\|\boldsymbol{x}\|_2^2 := \langle \boldsymbol{x}, \boldsymbol{x} \rangle = \boldsymbol{x}^\mathrm{T}\boldsymbol{x}$:

$$\widehat{P}(\lambda) = \underset{P(\lambda)\geq 0}{\operatorname{argmin}}\left(\frac{1}{2}\boldsymbol{P}^\mathrm{T}\boldsymbol{H}\boldsymbol{P} + \boldsymbol{f}^\mathrm{T}\boldsymbol{P}\right) \qquad \text{(Eq. 10)}$$

with $\boldsymbol{H} = \boldsymbol{A}^\mathrm{T}\boldsymbol{A} + \alpha^2\boldsymbol{L}^\mathrm{T}\boldsymbol{L}$ and $\boldsymbol{f}^\mathrm{T} = -\boldsymbol{A}^\mathrm{T}\boldsymbol{y}$. (The constant term $\boldsymbol{y}^T\boldsymbol{y}$ in Eq. 10 can be ignored for the arg-minimization). This quadric programming problem can be solved, i.e., by using the Matlab algorithm "quadprog" from the Optimization toolbox.

Beside the regularization in Eq. 4, we implemented an iterative Osher-Bregman iteration[31,32] as recommended in Ref. [21] in the context of the analysis of DEER data sets. This method is given by an iterative form[21,31]:

$$\widehat{\boldsymbol{P}}_{i+1}(\lambda) = \underset{\boldsymbol{P}(\lambda)\geq 0}{\operatorname{argmin}}\left(\frac{1}{2}\|\boldsymbol{A}\boldsymbol{P}-\boldsymbol{y}\|_2^2 + \frac{\alpha^2}{2}\|\boldsymbol{L}\boldsymbol{P}\|_2^2 + \langle \boldsymbol{P}, \boldsymbol{\phi}_i \rangle\right) \qquad \text{(Eq.11)}$$

with $\boldsymbol{\phi}_i = \boldsymbol{\phi}_{i-1} + \boldsymbol{A}^T(\boldsymbol{A}\widehat{\boldsymbol{P}}_i - \boldsymbol{y})$ and $\boldsymbol{\phi}_0 = \boldsymbol{0}$ as starting condition. The iteration stops when the standard deviation $\sigma(\boldsymbol{y} - \boldsymbol{A}\,\widehat{\boldsymbol{P}}_i)$ approximately reaches the noise level σ_N: $\left(\sigma(\boldsymbol{y} - \boldsymbol{A}\,\widehat{\boldsymbol{P}}_i) \approx \sigma_\mathrm{N}\right)$.

Model systems.

To determine the quality of the regularization, a number of theoretical model distributions and resulting EPR spectra were calculated. Three different models (model 1, model 2 and model 3) with arbitrary probability distributions $\boldsymbol{P}_\mathrm{Theo}(\lambda)$ were tested. We always used a multi-Gaussian distribution with up to seven independent Gaussian functions of different width and height (Supplementary Figure 1 and Supplementary Table 1). From these $\boldsymbol{P}_\mathrm{Theo}(\lambda)$ distributions, the respective X-band cw-EPR model spectra were calculated using the EasySpin algorithm "pepper"[1]. For each λ-value the cw-EPR spectrum $S(\lambda)$ was calculated using an $S = 3/2$ spin system with $D = 180$ GHz and $g_x = g_y = 2.00$ and $g_z = 2.03$, and a fixed line shape. λ was discretized in 667 steps. The microwave frequency was set to 9.4 GHz and each spectrum was simulated in a range from 30 to 550 mT with n = 8192 data points assuming a temperature of $T =$ 5 K. The theoretical spectrum is given by: $\boldsymbol{y}_\mathrm{Theo} = \sum_\lambda P_\mathrm{Theo}(\lambda)\boldsymbol{S}(\lambda)$. Noise was added afterwards to each theoretical spectrum. A $1/f$- and a Gaussian-noise model were used, each with

a signal-to-noise ratio (S/N) of 20 or 200. For regularization, the kernel matrix A (see Supplementary Figure 2 for EPR spectra of exemplary λ values) was calculated with the fictitious $S' = 1/2$ spin system of the lower Kramers doublet (Eq. 3). To compare the results of the regularization $\hat{P}$ with P_{Theo} the R^2 coefficient was used[21]:

$$R^2 = 1 - \frac{\sum_\lambda \left(P_{\text{Theo}}(\lambda) - \hat{P}(\lambda)\right)^2}{\sum_\lambda (\hat{P}(\lambda) - \langle \hat{P} \rangle)^2} \quad \text{(Eq. 12)}$$

Here, $\langle \hat{P} \rangle$ is the average of $\hat{P}(\lambda)$.

As described above, the α_{Opt} value was calculated by the L-curve criterion, the GCV, the rGCV, the AIC and the BIC methods. Additionally, all three regularization operators $\boldsymbol{L}_0, \boldsymbol{L}_1$ and $\boldsymbol{L}_2$ were tested. The regularization was performed with and without the Osher-Bregman iteration[22,31]. By comparing the regularization result $\hat{P}$ with the theoretical distribution P_{Theo}, a number of conclusions, mostly in line with published analyses of simulated DEER data sets[22], could be drawn:

First, the operators $\boldsymbol{L}_1$ and $\boldsymbol{L}_2$ gave comparable results, while the results using operator L_0 were significantly worse (Supplementary Figure 3).

Second, the GCV, the rGCV and the AIC methods (Eq. 7-9) were able to determine good values of α_{Opt}, while the L-curve method (Eq. 6) and the BIC method (Eq. 9) tended to lead to an over-smoothing of the results.

Finally, the Osher-Bregman iteration shows an improvement of the regularization result for a Gaussian noise model (Supplementary Figure 4), in particular for a low S/N of 20 (in line with reference [21]). However, the iteration showed more artifacts, if a $1/f$-noise model with a low S/N of 20 was used (Supplementary Figure 4).

Regularization and linewidth parameters.

So far, we assumed that the EPR spectra of nitrogenases are only dominated by an arbitrary distribution of the λ parameter, and have ignored other parameters that can influence the shape of an EPR spectrum. However, "intrinsic" line shapes may be affected, for example, by relaxation, field inhomogenities, non-resolved hyperfine couplings, and/or other small strains such as hyperfine- and/or g-strains. A simple method was implemented to take these influences into account: The kernel A is calculated for different intrinsic peak-to-peak line shapes (lwpp). Now the regularization is performed as described above for each kernel A_{lwpp} by solving:

$$\widehat{\boldsymbol{P}}(\lambda) = \underset{P(\lambda)\geq 0}{\operatorname{argmin}} \left(\frac{1}{2} \left\| \boldsymbol{A}_{\text{lwpp}} \boldsymbol{P} - \boldsymbol{y} \right\|_2^2 + \frac{\alpha^2}{2} \, \|\boldsymbol{LP}\|_2^2 \right) \quad \text{(Eq. 13)}$$

Afterwards, the graph

$$\rho(\text{lwpp}) = \log\left(\left\| \boldsymbol{A}_{\text{lwpp}} \widehat{\boldsymbol{P}} - \boldsymbol{y} \right\|_2^2 \right) \quad \text{(Eq. 14)}$$

is plotted against lwpp. The minimum of this function should represent the optimal intrinsic line shape. This procedure was tested using the calculated spectra of models 1, 2 and 3. All regularizations were

performed using operator L_2 without Osher-Bregman iteration, and α_{Opt} was determined by the GCV method. The parameter lwpp was discretized in 0.2 mT steps. Supplementary Figures 5 and 6 shows the $\rho(\mathrm{lwpp})$ curves for the case of an intrinsic Lorentzian linewidth of 1 mT, the respective probability distributions are depicted in Supplementary Figures 7 and 8, and the respective cw-EPR spectra in Supplementary Figures 9–12. This method provides very good results for high $\mathrm{S/N}$ of 200 and accurately reveals the chosen linewidth. Only at low signal-to-noise ratios ($\mathrm{S/N} = 20$) the minima of the $\rho(\mathrm{lwpp})$ curves become wider and shallower. As the experimental cw-EPR spectra analyzed in this study do have sufficiently high signal-to-noise ratios, this procedure is an easy and promising approach for determining the intrinsic linewidth.

Tikhonov regularization of selenium- and sulfur-labeled FeMo cofactors.

First, the cw-EPR spectrum of the resting state FeMo cofactor was simulated to determine the effective principal values of ${g'_x}^{1/2} = 3.664$, ${g'_y}^{1/2} = 4.309$, ${g'_z}^{1/2} = 2.012$. From these values the real principal values (g_x, g_y, g_z) were calculated by solving $Eq. 1$ and assuming $g_x = g_y$. By doing so, $g_x = g_y = 2.00$ and $g_z = 2.03$ were obtained in good agreement with literature values[12]. These values were kept fixed in all following calculations. The parameter λ was discretized in 667 steps $\left(0 \leq \lambda \leq \frac{1}{3}\right)$, and the resulting EPR spectrum was calculated for each λ value. For the $\rho(\mathrm{lwpp})$ graph, Lorentzian peak-to-peak linewidths were tested from 0.5 mT to 9 mT in 0.5 mT steps (Supplementary Figure 19).

As baseline artifacts can strongly influence the quality of the regularization, a baseline correction of all cw-EPR spectra was performed according to the following procedure. A cw-EPR spectrum of the buffer recorded under otherwise identical experimental conditions was subtracted from each cw-EPR spectrum. Afterwards, a polynomial baseline was manually subtracted and the resulting spectrum was integrated. From the integrated spectrum, a second baseline was subtracted and the spectrum was differentiated again. Before regularization, all spectra were truncated at $B_0 > 283$ mT as resonator background artifacts in the region of $g \approx 2$ reduced the quality of the regularization results, in particular at low microwave powers.

For regularization of experimental datasets, the GCV method was used for determining the α_{Opt} value. As a control, the α_{Opt} value was checked independently by visual inspection of the L-curve. $\boldsymbol{L_2}$ without Osher-Bregman iteration was used as regularization operator, as all cw-EPR spectra have a good S/N, so iteration hardly leads to improvements.

Grid-of-errors method.

Analysis of EPR spectra using the grid-of-errors method was first described by Azarkh and Groenen [8] and is used here to analyze our experimental EPR data sets via a second, independent method. The method was implemented as a Matlab algorithm and can be described as follows for two parameters a and b[8]: Given the spectrum $\boldsymbol{y}_{\mathrm{exp}}$, which can be described by a (unknown) density probability distribution

of two parameters a and b. The distribution is given by: $P(a,b) = P(a)P(b)$. Both parameters can be discretized (a in k steps and b in m steps), which leads to a probability distribution $P(i,j) = P_a(i)P_b(j)$ with $i \in \{1,\ldots,k\}$ and $j \in \{1,\ldots,m\}$ For each tuple (i,j) the EPR spectrum can be calculated as a $k \times m$ grid $G(i,j)$. An initial probability distribution $P_0(i,j) = P_{a,0}(i)P_{b,0}(j)$ is assumed, which can be a simple Gaussian distribution for each parameter. The first spectrum can now be calculated as

$$\boldsymbol{S}_0 = \sum_{i,j} \boldsymbol{G}(i,j)P_0(i,j) \qquad \text{(Eq. 15)}$$

with an error of

$$\chi^0 = \sqrt{\left(\boldsymbol{y}_{\text{exp}} - \boldsymbol{S}_0\right)^2}. \qquad \text{(Eq. 16)}$$

A refinement parameter c_0 is given. Now the grid-of-error is calculated as:

$$\chi_{i,j}^{\pm} = \sqrt{\left(\boldsymbol{S}_0 \pm c_0\boldsymbol{G}(i,j) - \boldsymbol{y}_{\text{exp}}\right)^2} \qquad \text{(Eq. 17)}$$

If $\chi_{i,j}^{\pm} < \chi^0$, an improvement of the fit is expected. In this case a new probability distribution is obtained: $P_1(i,j) = P_0(i,j) \pm c_0$. To ensure that the probability is positive, the subtraction is only done if $P_0(i,j) \geq c_0$. Afterwards the new spectrum

$$\boldsymbol{S}_1 = \sum_{i,j} \boldsymbol{G}(i,j)P_1(i,j) \qquad \text{(Eq. 18)}$$

and the new error

$$\chi^1 = \sqrt{\left(\boldsymbol{y}_{\text{exp}} - \boldsymbol{S}_1\right)^2} \qquad \text{(Eq. 19)}$$

are calculated. This process can be iterated until no significant improvement is achieved. The grid-of-error method can be extended to an arbitrary number of parameters.

We have applied the grid-of-error method to the same model spectra already analyzed with the regularization method to compare the results of the grid-of-error with the regularization for a single parameter distribution $P(\lambda)$ (Supplementary Figure 13). In this case, the grid G is identical to the kernel matrix A.

If $\chi^n < \chi^{n+1}$, c_0 is set to $c_0/2$ (start value $c_0 = 5 \cdot 10^{-4}$) to improve the refinement. The algorithm should stop if $\frac{|\chi^n - \chi^{n+1}|}{\chi^n} < \epsilon$ with $\epsilon = 10^{-6}$.

Grid-of-errors analysis of the selenium- and sulfur-labeled FeMo cofactors.

Cw-EPR spectra using the grid-of-error method were analyzed as follows: The parameter λ was discretized in 223 steps with $0 \leq \lambda \leq 1/3$. The parameter lwpp was discretized in 249 steps with $0 \leq \text{lwpp} \leq 25$ mT. For the initial probability distribution $P_0(i,j) = P_{0,\lambda}(i)P_{0,\text{lwpp}}(j)$ a Gaussian distribution was assumed for both $P_{0,\lambda}$ and $P_{0,\text{lwpp}}$. The experimental data sets were baseline-subtracted the same way as has been done using the regularization method.

Simulation software. All spectra simulations were performed by using the Easyspin algorithm "pepper" [1] together with Matlab 2019. The angle grid for the powder spectra was chosen so that no artifacts occur. The angle grid was usually set to 0.5°.

Multi-Gaussian fitting of the probability distributions of the Se-modified FeMo cofactor.

The results of the regularization of the measurements at: T = 5 K, P = 0.37 mW, modulation amplitude = 0.6 mT and an intrinsic linewidth of 2.5 mT (Figure 3, blue lines). A multi-Gaussian fit was used to calculate the areas of the individual species:

$$\mathrm{Gaussian}_n(x) = \sum_{k=1}^{n} w_k \frac{1}{\sqrt{2\pi\sigma_k^2}} \exp\left(-\frac{\left(x-x_{0,k}\right)^2}{2\sigma_k^2}\right) \qquad \text{(Eq. 20)}$$

In this case is the area of each Gaussian function: $A_i = w_k$. The total area is: $w_{\mathrm{tot}} = \sum_{k=1} w_k$. The results of the regularization and the corresponding multi-Gaussian fits are shown in Supplementary Figure 26 and Supplementary Table 2.

Supplementary References

1 Stoll, S. & Schweiger, A. EasySpin, a comprehensive software package for spectral simulation and analysis in EPR. *Journal of Magnetic Resonance* **178**, 42-55 (2006).

2 Lukoyanov, D. A. *et al.* Hydride conformers of the nitrogenase FeMo-cofactor two-electron reduced state $E_2(2H)$, assigned using cryogenic intra electron paramagnetic resonance cavity photolysis. *Inorganic Chemistry* **57**, 6847-6852 (2018).

3 Morrison, C. N., Spatzal, T. & Rees, D. C. Reversible protonated resting state of the nitrogenase active site. *Journal of the American Chemical Society* **139**, 10856-10862 (2017).

4 Hagen, W. R. Wide zero field interaction distributions in the high-spin EPR of metalloproteins. *Molecular Physics* **105**, 2031-2039 (2007).

5 Hagen, W. R., Hearshen, D. O., Harding, L. J. & Dunham, W. R. Quantitative numerical analysis of *g* strain in the EPR of distributed systems and its importance for multicenter metalloproteins. *Journal of Magnetic Resonance* **61**, 233-244 (1985).

6 Hearshen, D. O. *et al.* An analysis of *g* strain in the EPR of two [2Fe-2S] ferredoxins. Evidence for a protein rigidity model. *Journal of Magnetic Resonance* **69**, 440-459 (1986).

7 Froncisz, W. & Hyde, J. S. Broadening by strains of lines in the *g*-parallel region of Cu^{2+} EPR spectra. *The Journal of Chemical Physics* **73**, 3123-3131 (1980).

8 Azarkh, M. & Groenen, E. J. J. Simulation of multi-frequency EPR spectra for a distribution of the zero-field splitting. *Journal of Magnetic Resonance* **255**, 106-113 (2015).

9 Telser, J. EPR interactions – Zero-field splittings. *eMagRes* **6**, 207-233 (2017).

10 Hou, S. L., Summitt, R. W. & Tucker, R. F. Electron-paramagnetic-resonance and optical spectra of Cr^{3+} in SnO_2 single crystals. *Physical Review* **154**, 258-265 (1967).

11 Huynh, B. H., Münck, E. & Orme-Johnson, W. H. Nitrogenase XI: Mössbauer studies on the cofactor centers of the MoFe protein from *Azotobacter vinelandii* OP. *Biochimica et Biophysica Acta* **576**, 192-203 (1979).

12 Venters, R. A. *et al.* ENDOR of the resting state of nitrogenase molybdenum–iron proteins of *Azotobacter vinelandii*, *Klebsiella pneumoniae*, and *Clostridium pasteurianum*: ^{1}H, ^{57}Fe, ^{95}Mo, and ^{33}S studies. *Journal of the American Chemical Society* **108**, 3487-3498 (1986).

13 Hoffman, B. M., Weschler, C. J. & Basolo, F. The dioxygen adduct of *meso*-tetraphenylporphyrinmanganese(II), a synthetic oxygen carrier. *Journal of the American Chemical Society* **98**, 5473-5482 (1976).

14 Pilbrow, J. R. Effective *g* values for $S = 3/2$ and $S = 5/2$. *Journal of Magnetic Resonance* **31**, 479-490 (1978).

15 Phillips, D. L. A technique for the numerical solution of certain integral equations of the first kind. *Journal of the ACM* **9**, 84-97 (1962).

16 Chiang, Y.-W., Borbat, P. P. & Freed, J. H. The determination of pair distance distributions by pulsed ESR using Tikhonov regularization. *Journal of Magnetic Resonance* **172**, 279-295 (2005).

17 Ahmad, R., Vikram, D. S., Potter, L. C. & Kuppusamy, P. Estimation of mean and median pO_2 values for a composite EPR spectrum. *Journal of Magnetic Resonance* **192**, 269-274 (2008).

18 Twomey, S. On the numerical solution of the fredholm integral equations of the first kind by the inversion of the linear system produced by quadrature. *Journal of the ACM* **10**, 97-101 (1963).

19 Rein, S., Lewe, P., Andrade, S. L., Kacprzak, S. & Weber, S. Global analysis of complex PELDOR time traces. *Journal of Magnetic Resonance* **295**, 17-26 (2018).

20 Jeschke, G. *et al.* DeerAnalysis2006 – a comprehensive software package for analyzing pulsed ELDOR data. *Applied Magnetic Resonance* **30**, 473-498 (2006).

21 Ibáñez, L. F. & Jeschke, G. General regularization framework for DEER spectroscopy. *Journal of Magnetic Resonance* **300**, 28-40 (2019).

22 Edwards, T. H. & Stoll, S. Optimal Tikhonov regularization for DEER spectroscopy. *Journal of Magnetic Resonance* **288**, 58-68 (2018).

23 Tikonov, A. N. Solution of incorrectly formulated problems and the regularization method. *Soviet Mathematics Doklady* **4**, 1035-1038 (1963).
24 Tikhonov, A. N. Solution of incorrectly formulated problems and the regularization method. *Soviet Math. Dokl.* **4**, 1035-1038 (1963).
25 Hansen, P. C. Analysis of discrete ill-posed problems by means of the L-curve. *Society for Industrial and Applied Mathematics Review* **34**, 561-580 (1992).
26 Wahba, G. Practical approximate solutions to linear operator equations when the data are noisy. *SIAM Journal on Numerical Analysis* **14**, 651-667 (1977).
27 Golub, G. H., Heath, M. & Wahba, G. Generalized cross-validation as a method for choosing a good ridge parameter. *Technometrics* **21**, 215-223 (1979).
28 Lukas, M. A. Robust generalized cross-validation for choosing the regularization parameter. *Inverse Problems* **22**, 1883-1902 (2006).
29 Akaike, H. A new look at the statistical model identification. *IEEE Transactions on Automatic Control* **19**, 716-723 (1974).
30 Schwarz, G. Estimating the dimension of a model. *The Annals of Statistics* **6**, 461-464 (1987).
31 Osher, S., Burger, M., Goldfarb, D., Xu, J. & Yin, W. An iterative regularization method for total variation-based image restoration. *SIAM Multiscale Modeling & Simulation* **4**, 460-489 (2005).
32 Bregman, L. M. The relaxation method of finding the common point of convex sets and its application to the solution of problems in convex programming. *USSR Computational Mathematics and Mathematical Physics* **7**, 200-217 (1967).
